# Modeling changes in probabilistic reinforcement learning during adolescence

**DOI:** 10.1371/journal.pcbi.1008524

**Published:** 2021-07-01

**Authors:** Liyu Xia, Sarah L. Master, Maria K. Eckstein, Beth Baribault, Ronald E. Dahl, Linda Wilbrecht, Anne Gabrielle Eva Collins

**Affiliations:** 1 Department of Mathematics, University of California Berkeley, Berkeley, California, United States of America; 2 Department of Psychology, New York University, New York, New York, United States of America; 3 Department of Psychology, University of California Berkeley, Berkeley, California, United States of America; 4 School of Public Health, University of California Berkeley, Berkeley, California, United States of America; 5 Helen Wills Neuroscience Institute, University of California Berkeley, Berkeley, California, United States of America; Ecole Normale Superieure, FRANCE

## Abstract

In the real world, many relationships between events are uncertain and probabilistic. Uncertainty is also likely to be a more common feature of daily experience for youth because they have less experience to draw from than adults. Some studies suggest probabilistic learning may be inefficient in youths compared to adults, while others suggest it may be more efficient in youths in mid adolescence. Here we used a probabilistic reinforcement learning task to test how youth age 8-17 (N = 187) and adults age 18-30 (N = 110) learn about stable probabilistic contingencies. Performance increased with age through early-twenties, then stabilized. Using hierarchical Bayesian methods to fit computational reinforcement learning models, we show that all participants’ performance was better explained by models in which negative outcomes had minimal to no impact on learning. The performance increase over age was driven by 1) an increase in learning rate (i.e. decrease in integration time scale); 2) a decrease in noisy/exploratory choices. In mid-adolescence age 13-15, salivary testosterone and learning rate were positively related. We discuss our findings in the context of other studies and hypotheses about adolescent brain development.

## Author summary

Adolescence is a time of great uncertainty. It is also a critical time for brain development, learning, and decision making in social and educational domains. There are currently contradictory findings about learning in adolescence. We sought to better isolate how learning from stable probabilistic contingencies changes during adolescence with a task that previously showed interesting results in adolescents. We collected a relatively large sample size (297 participants) across a wide age range (8–30), to trace the adolescent developmental trajectory of learning under stable but uncertain conditions. We found that age in our sample was positively associated with higher learning rates and lower choice exploration. Within narrow age bins, we found that higher saliva testosterone levels were associated with higher learning rates in participants age 13–15 years. These findings can help us better isolate the trajectory of maturation of core learning and decision making processes during adolescence.

## Introduction

In the everyday world, perfectly predictable outcomes are rare. Yet, we need to track important events and their relationships to other events and actions. For example, we might want to learn where the best place to obtain food is, or where a potential mate likes to hang out—this might help us decide where to go, expecting a positive outcome to occur frequently, but not always. Our ability to learn about these probabilistic relationships is crucial for our daily life and decision making.

This challenge needs to be met by the developing brain, especially during adolescence [4–9]. Naively, one might assume that the brain simply gets better at this (and possibly all) forms of learning with brain maturation. However, what does *better* mean in this context? Most learning mechanisms are subject to tradeoffs between speed and stability. Fast learning may be suitable for a highly certain environment with deterministic relationships/statistics, but can lead to impulsive behavior in a more uncertain environment with probabilistic relationships/statistics [2, 10]. By contrast, slower learning that integrates over a longer time scale may lead to more robust and stable performance in probabilistic environments. During development, there may be periods where one form of learning is emphasized over the other. Changes could be gradual and monotonic, or show sharp steps when driven by factors such as hormonal changes at puberty onset [11]. There may also be inverted U shapes [3, 7, 12], that peak to support a sensitive period when specific information is available in the environment and/or when an organism needs to accomplish its transition to independence [13–15].

To study how learning changes across adolescence, we used the theoretical framework of reinforcement learning (RL). Computational RL models assume that we estimate the long-term values of an action in a given state by integrating over time the feedback we receive for choosing this action in this state, through a trial-and-error process [16]. RL has greatly enhanced our understanding of human behavior and the neural processes that underlie learning and decision-making in both certain and uncertain environments [17–21]. Moreover, RL processes offer a quantitative parameterization of individual differences: for example, RL decision noise may capture exploratory choice [4]; RL learning rates control the time scale of integration of rewards, with potential asymmetries between positive and negative outcomes [22, 23]; and RL forgetting parameters may capture memory dependent processes [12].

For these reasons, RL has been previously used to probe developmental changes in learning and decision making, including during adolescence [2–5, 12, 24, 25]. While there has been some consensus on certain developmental trends, such as lower decision noise with age [4], in general, developmental results in both how learning behavior changes and in how RL processes (and parameters) change are highly variable and dependent on the specific tasks used [4, 26].

To study how learning under uncertainty changes during adolescence, we used the *Butterfly task* [2], where participants needed to learn probabilistic associations that were stable throughout the task. We collected data from a sample of 297 participants across a wide age range (8–30), over-sampling participants age 8–18 to focus on the adolescent period (see S1 Fig for detailed breakdown of age group by sex). In fact, in this same sample, we conducted a total of four tasks that varied across multiple dimensions (such as deterministic/probabilistic feedback, stable/volatile contingencies, memory load, etc.), with the initial motivation to address the issue introduced by task heterogeneity [4]. However, the focus of this paper is on the Butterfly task alone. While we mainly present results from the Butterfly task, we also discuss comparisons and relationships with two other tasks in this sequence of four tasks [3, 12].

The Butterfly task tests participants’ ability to learn probabilistic associations between four butterflies and two possible preferred flowers from reward feedback. This task has been used in developmental studies before [2], and produced an intriguing result showing adolescent performance was greater than adults in a two group design (N = 41 adolescents age 13–17 and N = 31 adults age 20–30). We sought to further investigate performance in this task during development with a larger sample that would enable evaluation of the trajectory of development from age 8–30 and examine the role of puberty in changes in performance.

To evaluate the potential role of gonadal hormones and pubertal development in driving changes in learning, we also measured pubertal development and saliva testosterone (see Participants, S1 Text: Saliva collection and testosterone testing). We expected to observe an inverted U shape in performance that peaked in mid adolescence [27], coinciding with previously observed peaks in nucleus accumbens activation in response to rewarding outcomes [7, 28]. Previous studies have also found positive relationships between adolescent testosterone levels and nucleus accumbens activation [7, 29, 30]; therefore, we expected that pubertal development might explain the timing of any observed peak. A further motivation to conduct this study was to investigate the possibility that participants of different ages were differently sensitive to positive and negative outcomes, something that has been observed in other studies [31], but was not investigated previously using the Butterfly task [2].

Contrary to our predictions, we found no evidence for adolescent performance advantage in our version of the Butterfly task. Instead, we found performance increased through early adulthood, then stabilized. We used hierarchical Bayesian methods to fit computational RL models to the trial-by-trial data (see Hierarchical model fitting) and examined how participants integrated information across trials and made decisions. Increases in performance with age were explained by an increase in learning from rewarded outcomes and a decrease in exploration. These findings are largely consistent with studies of learning and decision making in other tasks that show steady improvement in performance across adolescent development [2, 4, 12]. We compare and contrast with findings that show adolescents outperforming adults [2, 3] to shed light on the conditions when adolescents vs. adults may show performance advantages in learning.

## Materials and methods

### Ethics statement

All procedures were approved by the Committee for the Protection of Human Subjects (CPHS number, community participants: 2016–06-8925; student participants: 2016–01-8280) at the University of California, Berkeley (UCB).

### Participants

A total of 297 (151 female) participants completed the task: 187 children and adolescents (age 8–18) from the community, 55 UCB undergraduate students (age 18–25), and 55 adults (age 25–30) from the community. Participants under 18 years old and their guardians provided their informed assent or written permission; participants over 18 provided informed written consent themselves.

We assessed biological sex (self-reported) and pubertal development for children and adolescents through saliva samples and through self-report with the pubertal development questionnaire, from which we calculated testosterone levels (T1, see S1 Text: Saliva collection and testosterone testing) and Puberty Development Score (PDS, [32]) respectively. The correlation between pubertal measures and age was very strong as expected (see S11 Fig).

Community participants were compensated with a $25 Amazon gift card for completing the experimental session, and an additional $25 for completing optional take-home saliva samples; undergraduate participants received course credit for participation. All participants were pre-screened for the absence of present or past psychological and neurological disorders.

### Experimental design

This task was the third in a sequence of four tasks that participants completed in the experimental session [12]. The task was a contextual two-armed bandit task with binary feedback: there were four stimuli (blue, purple, red, and yellow butterflies) and two bandits (pink and white flowers). Participants needed to figure out the preferred flower for each of the four butterflies through trial and error. Each butterfly had a preferred flower, which remained fixed throughout the experiment.

**Fig 1:**
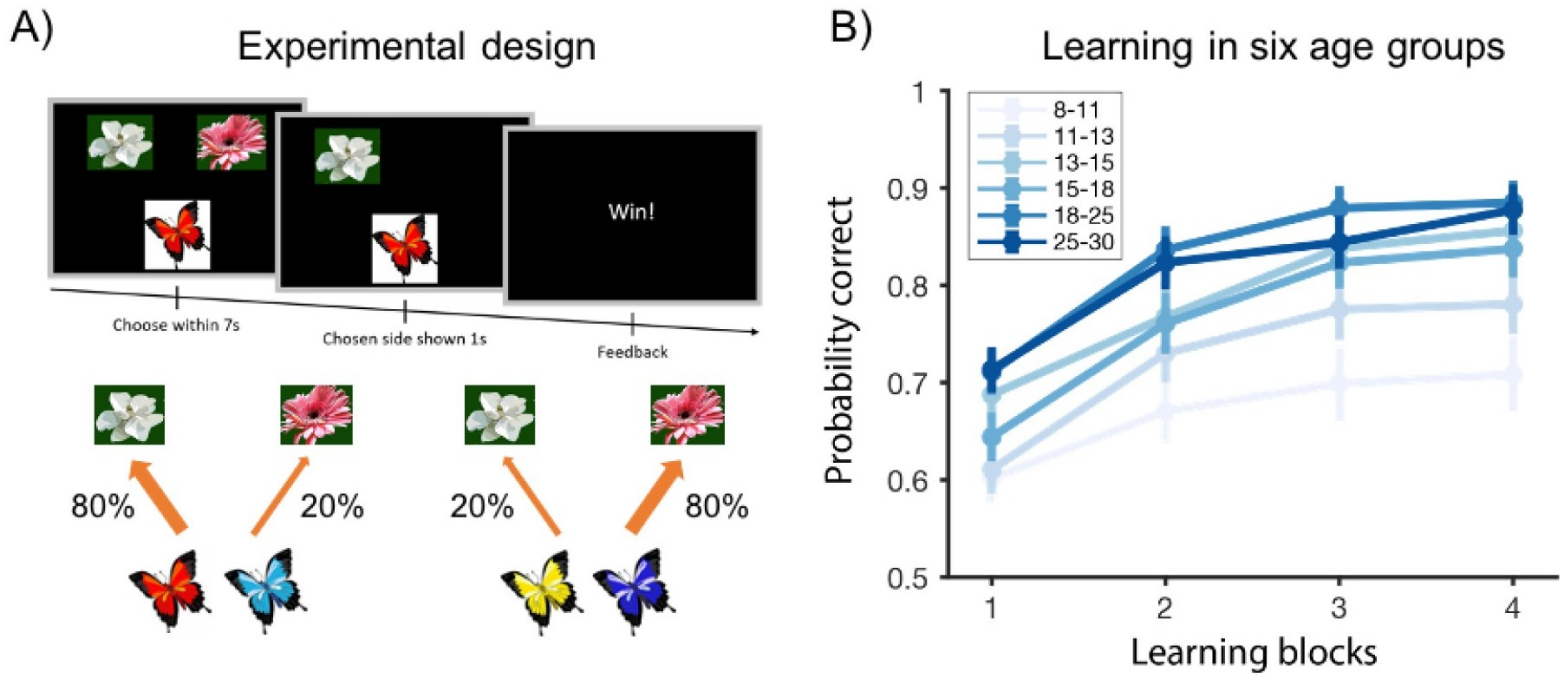
Experimental design and overall performance. (A) On each trial, participants needed to choose the butterfly’s preferred flower. Each butterfly’s preferred flower stayed the same throughout the experiment. If participants correctly picked the butterfly’s preferred flower, they observed a *Win!* feedback with 80% chance, and *Lose!* otherwise. The other choice delivered positive feedback only with 20% chance. Schematics were adapted from [2]. (B) Average probability of a correct choice over four 30-trial learning blocks. Learning curves showed all age groups learned the task, and that performance generally improved with age group.

On each trial (Fig 1A), participants were presented one butterfly and two flowers. They needed to choose a flower within 7s using a video game controller. They were instructed to respond as quickly as possible. The chosen flower would stay on the screen for 1s. If participants correctly chose the preferred flower, they would receive positive feedback (*Win!*) with 80% chance; however, 20% of the time the other flower would be the rewarding one, resulting in negative feedback (*Lose!*). The feedback stayed on the screen for 2s. If participants received the ‘Win!’ feedback, they received 1 point, whereas ‘Lose!’ meant 0 points. Participants were instructed to obtain as many points as possible and they could always see the total number of points earned so far on the upper right corner of the screen. The total amount of points won was not translated to a real-life reward such as money. There were 30 trials for each butterfly, resulting in a total of 120 trials. The butterfly-flower mapping, position of flowers, sequence of butterflies and the probabilistic feedback were pre-randomized and counterbalanced across participants.

### Exclusion criteria

One participant under 18 was excluded because only 18 out of 120 trials were completed, resulting in 296 participants. We also excluded participants who were overall more likely to change their choice of flower for a given butterfly (switch) than repeat it after receiving positive feedback, which suggested that they either did not understand the task or were not engaged in it. 20 participants under 18 and one undergraduate participant were excluded due to this criterion. Note that all behavioral results presented later in Overall performance, Reaction time, and Mixed-effect logistic regression hold with the original sample of 296 participants.

To further conservatively identify participants who were not engaged in the task, we excluded participants who had worse than chance performance and satisfied one of the following “low data quality” criteria: (1) high proportion of trials where the participant picked the same choice as the previous trial, (2) high proportion of trials where the participant changed their choice, (3) presence of too long sequences of trials where the participant kept choosing the same flower, and (4) high proportion of missing trials. All of those criteria were determined by elbow points (see S1 Text: Exclusion criteria details), and indicated a lack of reactivity to the task’s inputs. Applying these *conjunctive* criteria resulted in further exclusion of 11 participants (S1 Table), bringing the total number of *on task* participants for later analysis to 264 (138 female), with 157 participants under 18 (see S1 Table for breakdown of exclusion by age). All behavioral and modeling results presented later in Results hold with weaker exclusion criteria (i.e. with the sample of 275 participants).

### Model-independent analysis

For each participant in each trial, we recorded whether they chose the butterfly’s preferred flower (*correct* choice or not), and whether they received reward or not (win vs. lose), which was different due to the probabilistic nature of the task. As an aggregate measure of performance, we computed average accuracy within each of the four 30-trial learning blocks for each participant. We also computed median and standard deviation of reaction time within each learning block. We ran (linear and quadratic) regression to assess whether those behavioral metrics changed with age and pubertal measures.

We also calculated the proportion of trials (*p*) among all 120 trials where participants correctly chose each butterfly’s preferred flower as an overall performance measure. Because this proportion was not normally distributed across participants (Kolmogorov–Smirnov test, *p* = 0.003), we instead used log odds ($\text{log}\frac{p}{1-p}$) for all later statistical tests. The log odds were normally distributed (Kolmogorov–Smirnov test, *p* = 0.26).

We used the median reaction time for each participant as a speed measure. Because reaction time was not normally distributed across participants (Kolmogorov–Smirnov test, *p* = 0.02), for all later statistical tests, we used log-transformed reaction time, which was normally distributed (Kolmogorov–Smirnov test, *p* = 0.8).

**Fig 2:**
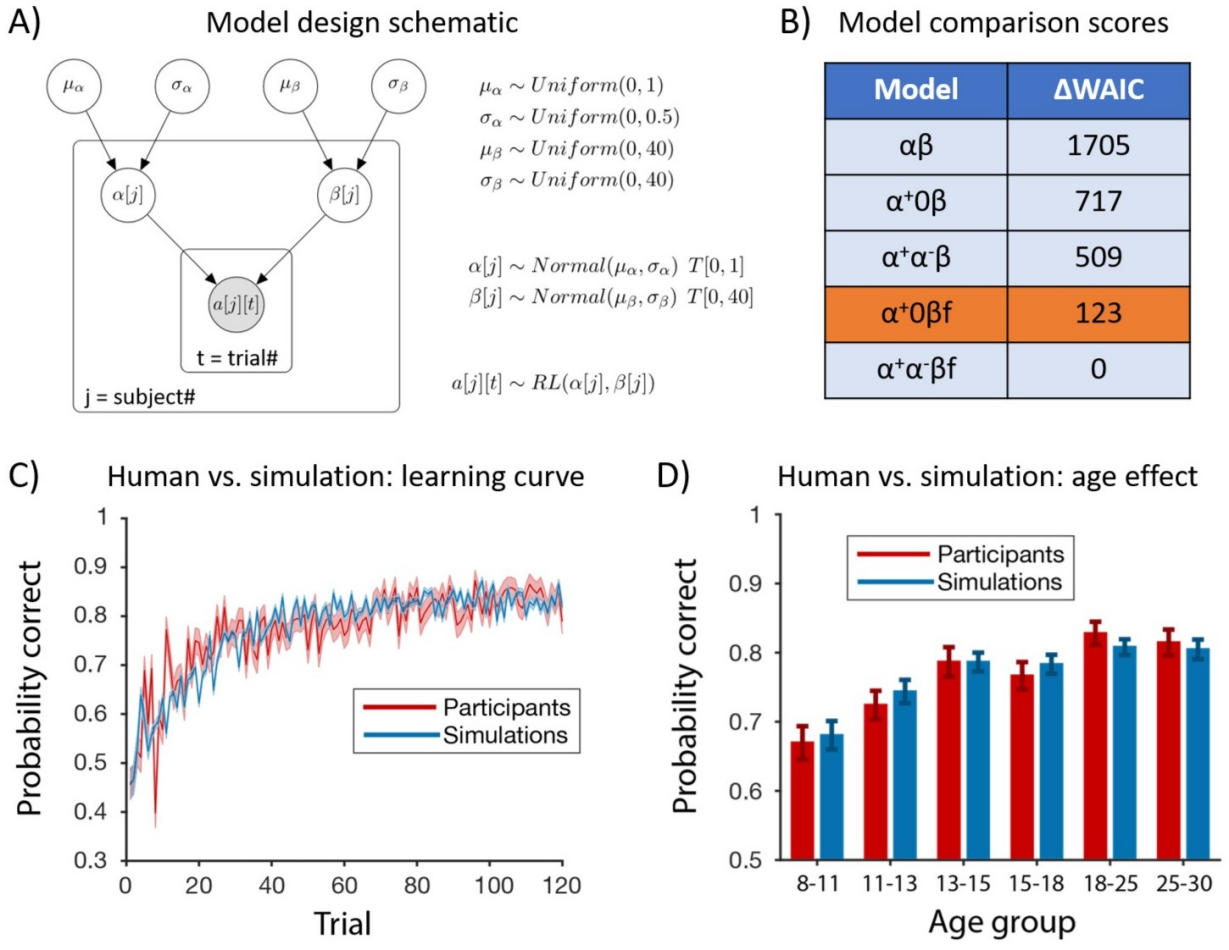
Hierarchical Bayesian modeling and model comparison. (A) We illustrate the hierarchical model design with the *αβ* model as an example, We assumed that parameters of individual participants came from the same group level distribution, which is truncated normal parametrized by the group-level mean and standard deviation (*μ*_*α*_ and *σ*_*α*_ for *α*; *μ*_*β*_ and *σ*_*β*_ for *β*). The group-level mean and standard deviation followed weakly informative priors (uniform and bounded). The parameters for each individual participant are used in the likelihood of each action on every trial based on the *αβ* model. *T*[*m*, *n*] indicates truncation of distribution (e.g. the learning rate, *α*, is bounded by [0, 1]). The filled circle represented observed variable (in this case, participants’ choices on each trial); unfilled circles represented latent variables (in this case, group and individual model parameters). (B) We calculated WAIC for model comparison. ΔWAIC represents the difference between the WAIC of the considered model and the model with the lowest (i.e. best) WAIC. While the *α*^+^*α*^−^*βf* model had the lowest WAIC score, the *α*^−^ parameter was not recoverable and showed signs of overfitting (see S1 Text: Model comparison extended). We thus focus on the *α*^+^0*βf* model. (C-D) We used fitted parameters from the *α*^+^0*βf* model to generate simulated trajectories. The simulated performance captures the average learning curve throughout the experiment (C), and replicates the age effect (D).

To visualize age effects (Figs 1 and 2), we broke participants under age 18 into four equal-size groups within each sex respectively, and then combined both sexes (see S1 Text: Pubertal effects extended). The boundaries for the four age groups were approximately 8–11, 11–13, 13–15, and 15–18 (the exact boundaries for each age group and sex can be found in S2 Table). Together with two age groups above 18 (18–25 for students and 25–30 for community participants), we had a total of six age groups.

Going beyond aggregate measures across trials, we also ran a mixed effect logistic regression to predict participants’ choices on a trial-by-trial basis and tested how previous reward history and delay affected learning and decision making. Specifically, for each trial, we defined the *reward history*, *r*, as the number of trials that participants had previously received a *Win!* feedback for the current butterfly; we also defined *delay*, *d*, as the number of intervening trials since the last time the participant encountered the same butterfly and got rewarded. We then used the lme4 package in R to test *p*(*correct*) = *logit*(1 + *r* + *d* + (1+ *r* + *d*|*sub*)), where *sub* represented random effects of individual participants respectively. All regressors were z-scored.

We analyzed whether the random effects and other behavioral measures varied with age using linear and quadratic regressions. While quadratic regressions could provide initial evidence for nonlinear relationships, to ascertain true (inverse) U shapes, we used two-line regression [33]. Two-line regression detects a sign change of the regression slopes for low and high values of age respectively, without assuming quadratic as the true relationship between age and the dependent variables.

Using multiple linear regressions and ANOVA tests, we also assessed the effect of sex as a way to control for a potential, known source of variance and thus improve the generalizability of our findings [34]. Note that this is particularly important when assessing age and puberty effects during adolescence, as puberty has known different timelines across sexes, and different hormonal markers (also visible in our sample).

### Computational models

While the logistic regression described above (Model-independent analysis) could serve as a descriptive model for participants’ trial-by-trial choices, we also used computational RL modeling to obtain a more quantitative and mechanistic understanding of participants’ trial-by-trial learning. We applied six variants of RL models, then used the parameter estimates of the best fitting model as the basis for inference.

#### Classic RL (*αβ*)

Our simplest RL model was the *αβ* model, with just two free parameters, *α* (learning rate) and *β* (inverse temperature). The *αβ* model used Q-learning to compute *Q*(*b*, *a*), as the expected value of choosing flower *a* for butterfly *b*. On trial *t*, the probability of choosing *a* was computed by transforming the Q-value with a softmax function:

$$\begin{array}{c} P\left(a|b\right)=\frac{exp(\beta Q_{t}\left(b,a\right))}{\sum_{i=1}^{2}exp\left(\beta Q_{t}\left(b,a_{i}\right)\right)}, \end{array}$$ (1)

where *Q*_*t*_(*b*, *a*) was the Q-value until trial *t*. The inverse temperature parameter *β* thus controls how exploratory/stochastic the decision making process is, with higher *β* resulting in more deterministic choices. After observing reward *r*_*t*_ (0 for “Lose!” or 1 for “Win!”), the Q-value *Q*(*b*, *a*) was updated through the classic delta rule:

$$\begin{array}{c} Q_{t+1}\left(b,a\right)=Q_{t}\left(b,a\right)+\alpha RPE, \end{array}$$ (2)

where *RPE* = *r*_*t*_ − *Q*_*t*_(*b*, *a*) is the reward prediction error. Note that this delta rule can also be rewritten as:

$$\begin{array}{c} Q_{t+1}\left(b,a\right)=\left(1-\alpha \right)Q_{t}\left(b,a\right)+\alpha r_{t}, \end{array}$$ (3)

which shows the updated Q-value (*Q*_*t*+1_) as a linear combination of past estimates (*Q*_*t*_) and the most recent reward (*r*_*t*_). Thus, the learning rate parameter *α* is often interpreted as a time integration constant, controlling how much of the past estimate contributes to the current estimate. For example, *α* = 1 would result in one-shot learning, i.e. set Q-value to be identical to the reward feedback each trial, resulting in an integration time scale of one trial (and no information about any other past trials). Smaller *α* results in integrating reward information across more trials from present into the past. Note that this time integration constant occurs only over trials in which the specific stimulus is present, rather than all trials.

We initialized all Q-values to the uninformative value of 0.5 (the average of positive and negative feedback) for this model and all other models under consideration.

#### RL with asymmetric learning rates (*α*^+^*α*^−^*β*)

The *α*^+^*α*^−^*β* model differed from the *αβ* model by using two distinct learning rate parameters, *α*^+^ and *α*^−^. Recent literature suggests that humans learn from positive and negative feedback to different degrees, and even with potentially different neural mechanisms [23]. RL models with asymmetric learning rates have also been widely used and examined in theoretical [22, 35] and developmental [1, 24, 31, 36, 37] contexts, especially in studies with probabilistic tasks.

Having both *α*^+^ and *α*^−^ allowed the model to have different sensitivity to positive and negative RPE [38]. Specifically, in Eq 2, *α*^+^ was used when *RPE* > 0, and *α*^−^ otherwise.

#### Asymmetric RL with *α*^−^ = 0 (*α*^+^0*β*)

The *α*^+^0*β* model was the same as the *α*^+^*α*^−^*β* model, except that the *α*^−^ parameter was set to 0. This change made the model insensitive to negative feedback. We included this model because of the observation that the fitted values of the *α*^−^ parameter from the *α*^+^*α*^−^*β* model were very small and not recoverable (see S1 Text: Model comparison extended).

#### RL with forgetting (*αβf*)

The *αβf* model builds upon the *αβ* model by including an additional forgetting parameter, *f*. On each trial, after applying the delta learning rule Eq 2, Q-values decay toward the uninformative starting value of 0.5, implementing a forgetting process:

$$\begin{array}{c} Q_{t+1}\left(b,a\right)=\left(1-f\right)*Q_{t+1}\left(b,a\right)+f*0.5. \end{array}$$ (4)

Eq 4 is implemented for all butterfly-flower pairs except the butterfly and the selected flower on the current trial. Note that forgetting thus occurs on every trial (in contrast to integration via learning rate which occurs only on stimulus-specific trials).

#### Asymmetric RL with forgetting (*α*^+^*α*^−^*βf*)

The *α*^+^*α*^−^*βf* model has both asymmetric learning rates for positive and negative feedback and the forgetting parameter.

#### Asymmetric RL with *α*^−^ = 0 and forgetting (*α*^+^0*βf*)

For factorial design, we included the *α*^+^0*βf* model, which builds upon the *α*^+^*α*^−^*βf* model by setting *α*^−^ = 0.

### Hierarchical model fitting

We fitted all RL models using hierarchical Bayesian methods [39] jointly to all participants, instead of to each participant independently. To illustrate the hierarchical model design (Fig 2A), we use the simplest model, *αβ*, as an example. We specified weakly informative priors for the mean and standard deviation of the group-level learning rate (*μ*_*α*_ and *σ*_*α*_) and the group-level inverse temperature (*μ*_*β*_ and *σ*_*β*_). We assumed that these group-level parameters were all uniformly distributed over the natural ranges of the parameters (for example, we truncated *μ*_*α*_ at 0 and 1, since we know the learning rate parameter is between 0 and 1). We then assumed that the parameters for each participant were drawn from a prior distribution defined by the group-level parameters: for example, *α*[*j*] for participant *j* was drawn from a normal distribution *Normal*(*μ*_*α*_, *σ*_*α*_) truncated at 0 and 1. Individual participants’ parameters were then used in the likelihood of each participant’s actions on each trial (*a*[*j*][*t*]) according to the *αβ* model, where *j* and *t* indicate participant number and trial number, respectively.

The hierarchical model made the likelihood intractable [40], but it can be well approximated by sampling. We used No-U-Turn sampling, a state-of-the-art Markov Chain Monte Carlo (MCMC) algorithm implemented in the probabilistic programming language Stan [41], to sample from the joint posterior distribution of model parameters for all participants. Compared to the classic participant-wise maximum likelihood estimation approach, hierarchical model fitting with MCMC provides more stable point estimates for individual participants and allows natural inference of effects on parameters at the group level [42].

For each model, we ran 4 MCMC chains, with each chain generating 4000 samples (after 1000 warmup samples), resulting in 16000 samples per model for later inference. We assessed convergence for all models using the *matstanlib* library [43]. In particular, we ensured that $\hat{R}$ statistics for all free parameters were below 1.05; that the effective sample sizes (ESS) for all free parameters were more than 25 × the number of chains; and that samples generally were not the result of divergent transitions. Note that these criteria are more stringent than the standard criteria for convergence of $\hat{R}\leq 1.1$ and ESS > 5× number of chains as per [39]. Among all 6 models, only the *αβf* model was unable to converge when fitted hierarchically, thus we fitted the *αβf* model independently for each participant. The results presented later for the *αβf* model all came from non-hierarchical fitting. We also fitted the other 5 models non-hierarchically in order to compare with the *αβf* model, and summarized the results in the supplement (S6 Fig).

Hierarchical Bayesian modeling also provides a natural way to test for potential age effects on model parameters. Specifically, we incorporated the regression model using age to predict model parameters into the original graphical model (Fig 2A), and directly sampled regression coefficients for age jointly with other model parameters.

Same as before, we assumed that the model parameters for individual participants followed a truncated normal distribution with a group-level standard deviation, but now we replace the prior on the group-level mean with a regression statement with respect to age. For example, to probe linear effects of age on *α*, we assumed that the parameter *α*[*j*], used to compute the likelihood of participant *j*’s choices, followed *Normal*(*α*_*intercept*_ + *α*_*linear*_ * *age*[*j*], *σ*_*α*_), *T* [0, 1], where *age*[*j*] was the z-scored age of participant *j*, and *α*_*intercept*_, *α*_*linear*_ were regression coefficients for which we set weakly informative priors. To probe quadratic effects, we just further included *α*_*quadratic*_ * *age*[*j*]^2^.

To test for effects of age on the model parameters, we examined whether the posterior distribution of all 16000 samples for the linear (*α*_*linear*_) and quadratic (*α*_*quadratic*_) regression coefficients were significantly different from 0.

### Parameter and model identifiability and validation

We verified that model parameters and models themselves were identifiable using generate and recover procedures (see S1 Text: Model comparison extended, [44]). We validated models by simulating models with fitted parameters 100 times per participants, and comparing model simulations with behavior (S4 Fig).

## Results

### Overall performance

To assess learning progress and potential age effects, we first calculated the proportion of correct trials within each of the four 30-trial learning blocks for 6 age groups (Fig 1B). As indicated in S1 Text: Pubertal effects extended, we grouped all participants under 18 into four equal-sized bins (N = 39, 39, 39, 40). The other two groups were undergraduate participants (age 18–25, N = 53) and adult community participants (age 25–30, N = 54).

All age groups exhibited learning over the course of the experiment. Specifically, we found a significant main effect of age group and block on participants’ performance (two-way mixed-effects ANOVA, age group: *F*(5, 255) = 8.5, *p* < 0.0001; block: *F*(3, 765) = 136, *p* < 0.0001). There was no interaction between age group and block (two-way mixed-effects ANOVA: *F*(15, 765) = 1, *p* = 0.49). This shows that participant’s performance improved as the experiment progressed, and older participants generally outperformed younger participants.

To further characterize the effect of age on overall performance, we computed the proportion of correct trials over all 120 trials. We found that the overall performance of 13–18 year-olds (top two quartiles) was significantly higher than of 8–13 year-olds (bottom two quartiles; unpaired t-test, *t*(1, 155) = 3.5, *p* = 0.0001), and significantly lower than of 18–25 year-olds (unpaired t-test, *t*(1, 130) = 2.5, *p* = 0.01). However, there was no significant difference (unpaired t-test, *t*(1, 105) = 0.2, *p* = 0.8) between the performance of 25–30 year-olds and 18–25 year-olds (Figs 1B and 2D).

**Fig 3:**
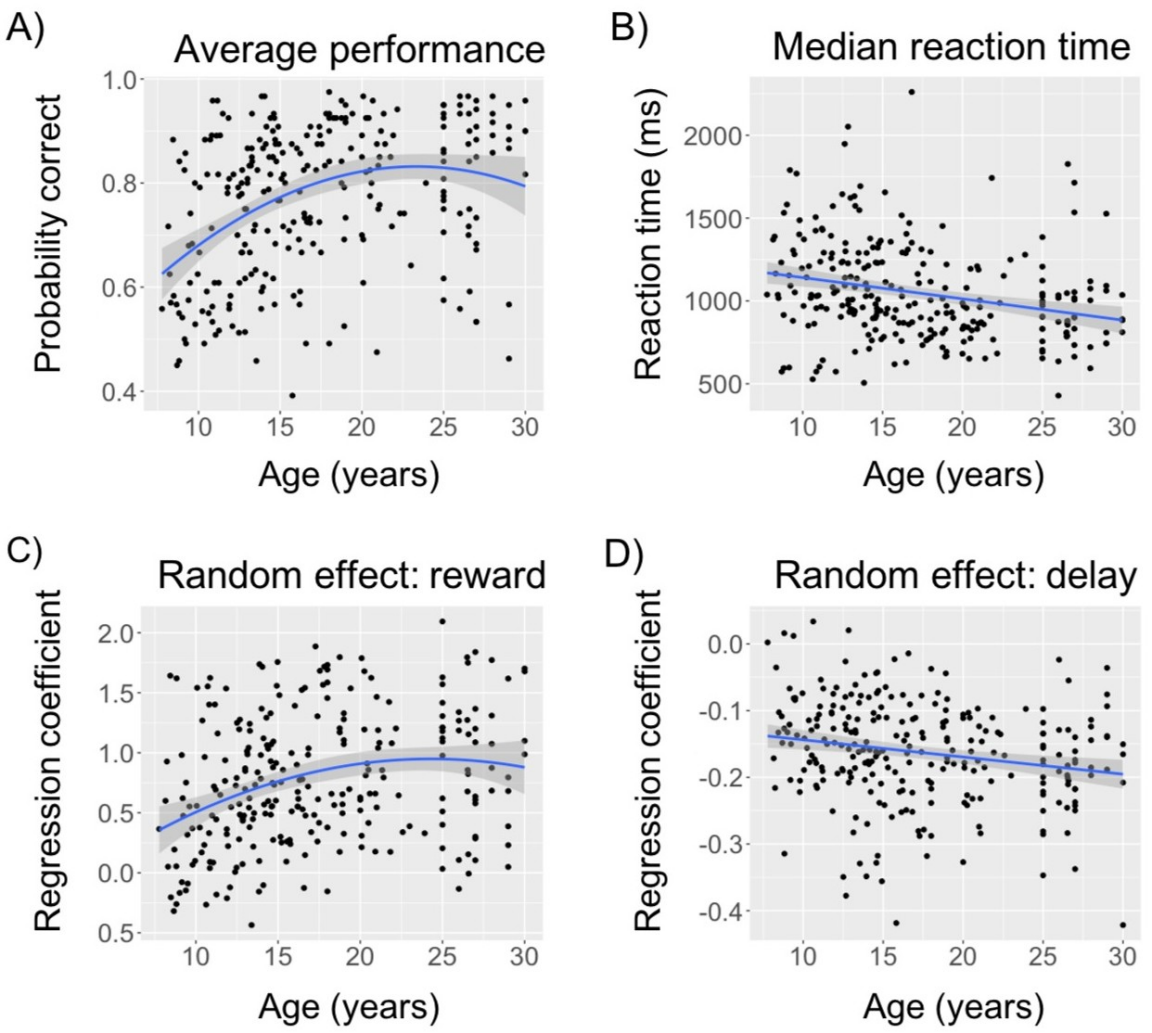
Age effects on participants’ behavior. Scatter plot of age (x-axis) and (A) probability of choosing the correct response, (B) median reaction time, (C) random effect for reward history, and (D) random effect of delay. Each black dot represents one participant. The blue curve represents linear/quadratic regression line. There was no effect of sex in any analysis. Shaded region represents 95% confidence interval.

To examine the continuous relationship between participants’ performance and age, we ran a regression analysis using age to predict performance (Fig 3A). We found that including a quadratic term improved model fit (sequential ANOVA: *F*(1, 261) = 7.6, *p* = 0.006). The regression analysis revealed linear and quadratic effects of age on performance (linear: *β*_*age*_ = 0.05, 95% CI = [0.03, 0.07]; quadratic: *β*_*age*^2^_ = −0.004, 95% CI = [−0.007, −0.001]). There was no effect of sex or its interaction with age (multiple linear regression, both *p*’s > 0.45).

To identify whether the quadratic effect was indicative of an inverse U shape, we conducted the two-line regression [33]. We found the break point at around 19 years old, before which *β*_*age*_ = 0.11, *z* = 5.24, *p* < 0.0001, and after which *β*_*age*_ = 0, *z* = 0.14, *p* = 0.9. This indicates that performance linearly increased with age through adolescence and stabilized in early adulthood, showing that the quadratic effect reflected a linear increase then stable process rather than an inverse U-shape.

### Reaction time

We also computed the median (Fig 3B) and standard deviation of reaction time for each participant. We found a linear effect of age on median reaction time (*β*_*age*_ = −0.01, 95% CI = [−0.02, −0.006]). This suggests that participants reacted faster with age, confirming previous results [12]. Adding a quadratic term did not improve model fit (sequential ANOVA: *F*(1, 261) = 1, *p* = 0.3). We also found a linear effect of age on the standard deviation of reaction time (linear regression: *β*_*age*_ = −0.02, 95% CI = [−0.03, −0.01]); adding a quadratic term improved model fit (sequential ANOVA: *F*(1, 261) = 17, *p* < 0.0001; quadratic regression: *β*_*age*^2^_ = 0.003, 95% CI = [0.002, 0.005]). Two-line regression revealed a break point at 19 years old, before which we found *β*_*age*_ = −0.05, *z* = −4.15, *p* < 0.0001, and after which we found *β*_*age*_ = 0.03, *z* = 1.88, *p* = 0.06. This indicates that the variability in reaction time decreased with age, and this decrease stabilizes in early adulthood and might even invert, consistent with previous findings [12, 45]. There was no significant effect of sex on the median reaction time (unpaired t-test, median: *t*(1, 262) = 0.4, *p* = 0.7), but female participants had a significantly smaller standard deviation than male participants (unpaired t-test: *t*(1, 262) = 2.72, *p* = 0.0085).

These results indicate better performance and faster responses in older participants, ruling out speed-accuracy tradeoffs. Both age group (Fig 1B) and continuous age (Fig 3A) analyses revealed a nonlinear saturating relationship between age and performance.

### Mixed-effect logistic regression

To better probe trial-by-trial learning dynamics, we used reward history and delay to predict the probability of a correct choice on each trial in a mixed-effect logistic regression. We found significant fixed effects of reward history and delay (*β*_*r*_ = 0.8, *β*_*d*_ = −0.17, both *p*’s < 0.0001). This suggests that participants were more likely to pick the preferred flower as they received more reward feedback for the butterfly (reinforcement learning effect), and encountered the butterfly more recently (forgetting effect).

We found linear and quadratic effects (linear: *β*_*age*_ = 0.02, 95% CI = [0.01, 0.03]; quadratic: *β*_*age*^2^_ = −0.002, 95% CI = [−0.004, −0.0003]; sequential ANOVA: *F*(1, 261) = 5, *p* < 0.02) of age on the random effect of reward history (Fig 3C). Two-line regression reveals a break point at around 21 years old, before which we found *β*_*age*_ = 0.04, *z* = 3.84, *p* = 0.0001, and after which we found *β*_*age*_ = 0.02, *z* = 0.75, *p* = 0.45. Therefore, similar to the trend we observed for overall performance (Fig 3A), participants’ sensitivity to reward increased with age and stabilized in early adulthood. We also found that participants became more sensitive to delay with age, shown by the linear effect (*β*_*age*_ = −0.003, 95% CI = [−0.004, −0.001]) of age on the random effect of delay (Fig 3D). Adding a quadratic term did not improve model fit (sequential ANOVA, *F*(1, 261) = 3, *p* = 0.08).

### Computational modeling

We used computational modeling and model comparison to obtain a mechanistic understanding of participants’ trial-by-trial learning and decision making. We fitted all participants jointly using hierarchical Bayesian modeling [39] combined with sampling [41] for approximating the likelihood function (see Hierarchical model fitting).

#### Model comparison

We used WAIC to compare the relative fit of models at the population level [46], an information criterion that penalizes model complexity appropriately for hierarchical Bayesian models. WAIC is fully Bayesian and invariant to reparametrization. Smaller WAIC indicates a better fit to the data, controlling for complexity. Since the *αβf* model was unable to converge when fitted hierarchically, we compared the other five hierarchical models using WAIC. Model comparison results for all six models fitted non-hierarchically with BIC can be found in the supplement (S6 Fig).

The *α*^+^*α*^−^*βf* model with asymmetric learning rates and the forgetting parameter had the lowest (best) WAIC score (Fig 2B). However, a generate and recover procedure [44] showed that the *α*^−^ parameter values in the *α*^+^*α*^−^*βf* model were very close to 0 (see S1 Text: Model comparison extended), and that they were not adequately recoverable (and therefore unsuitable to use as the basis for inference, see S2A and S3 Figs). Consequently, we focus on the model with the next best WAIC score, *α*^+^0*βf*, which could be successfully recovered from (see S1 Text: Model comparison extended), for further analysis. Note that conclusions for the *α*^+^, *β*, and *f* parameters remain the same if we used the *α*^+^*α*^−^*βf* model instead. Furthermore, the *α*^−^ parameters were generally very small for the fitted *α*^+^*α*^−^*βf* model (S2A Fig). This suggests that participants were learning either very little from negative feedback or not at all. Moreover, the *α*^+^0*βf* model resulted in better model validation (see S1 Text: Model comparison extended, S4 Fig), suggesting that *α*^+^*α*^−^*βf* at the population level might be overfitting.

We validated the best-fitting model, *α*^+^0*βf*, by simulating synthetic choice trajectories from fitted parameters (i.e., by generating posterior predictive distributions; Fig 2C and 2D) [47]. Model simulations captured the average learning curve throughout the entire experiment (Fig 2C) and age effects on overall performance (Fig 2D).

#### Age differences in model parameters

With the winning model *α*^+^0*βf*, we next asked which computational processes drove the changes in performance over age by testing how model parameters changed with age. We adapted hierarchical Bayesian modeling to probe effects of age on model parameters. Specifically, we incorporated the regression of age as a predictor of model parameters into the hierarchical Bayesian model (Fig 2A), and directly sampled regression coefficients for age jointly with other model parameters (see Hierarchical model fitting).

**Fig 4:**
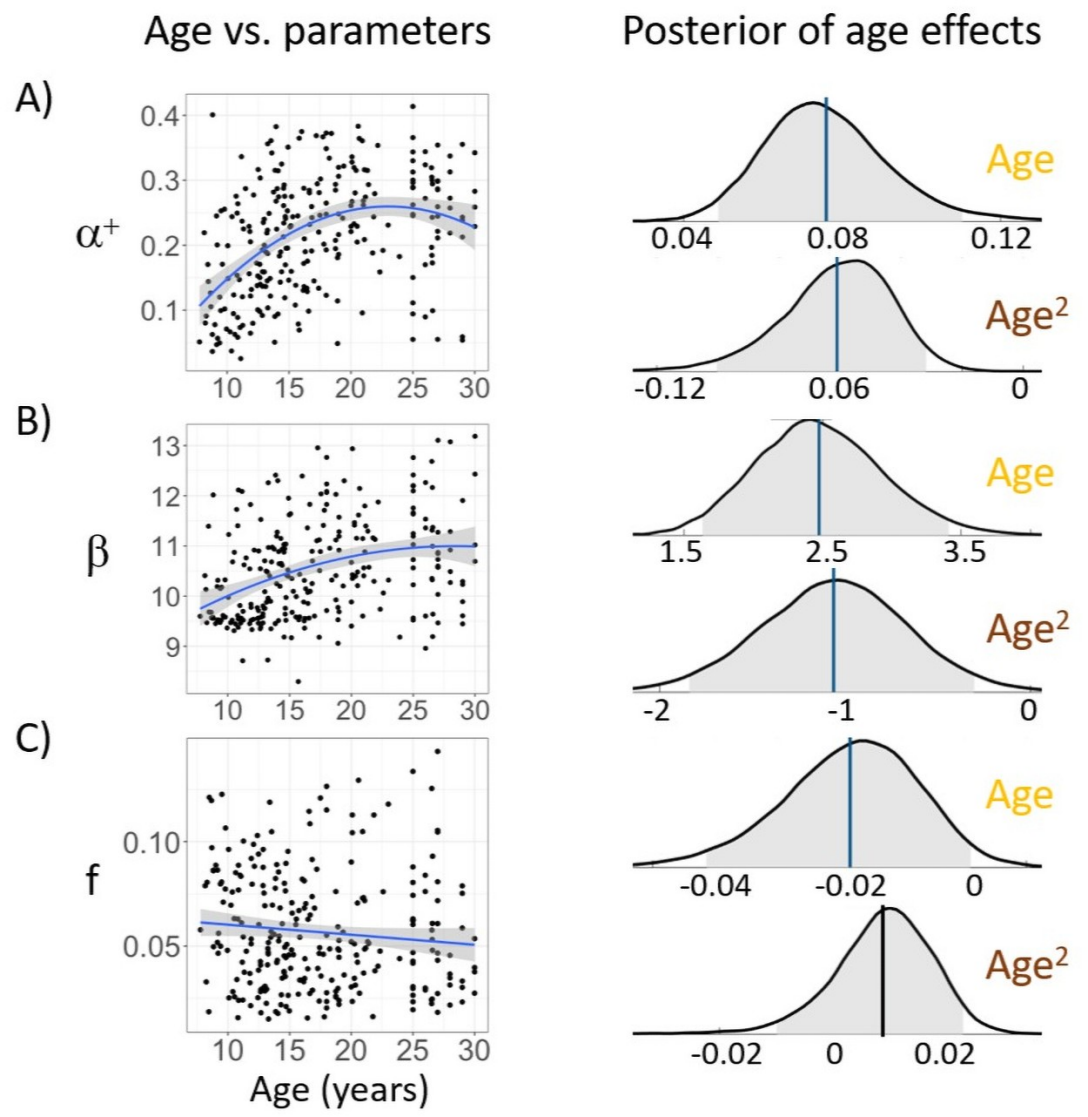
Age effects on model parameters. We directly incorporated age-related parameters into MCMC sampling to test within the hierarchical Bayesian modeling framework whether age had a linear or quadratic effect on all three model parameters: *α*^+^ (A), *β* (B), *f* (C). Left panel: individual parameters from the original *α*^+^0*βf* model plotted against age. For visualization, we included a regression line; the shaded region indicates 95% CI. Right: distribution of posterior samples for linear (top, yellow) and quadratic (bottom, brown) regression coefficients. The vertical line represents the mean of all samples, with blue indicating an effect being present (i.e., 95% CI not including 0), and black indicating no effect. Shaded region shows 95% confidence interval.

To test for effects of age on the model parameters, we examined whether the 95% credible interval (CI) of the posterior samples for each of the linear and quadratic regression coefficients did or did not include 0, where 0 indicates no effect (Fig 4). We found linear and quadratic effects of age on *α*^+^ (linear coefficient 95% CI = [0.05, 0.11]; quadratic coefficient 95% CI = [−0.1, −0.03]) and *β* (linear CI = [1.6, 3.4]; quadratic CI = [−1.9, −0.3]). The trajectory of quadratic change over age for *α*^+^ and *β* closely mimicked that for overall performance (Fig 3A). We also found marginally linear (Fig 3C), but not quadratic effects of age on *f* (linear CI = [−0.04, 0.001], *p* = 0.066; quadratic CI = [−0.01, 0.02]), with the forgetting parameter potentially decreasing over age.

### Pubertal effects

**Fig 5:**
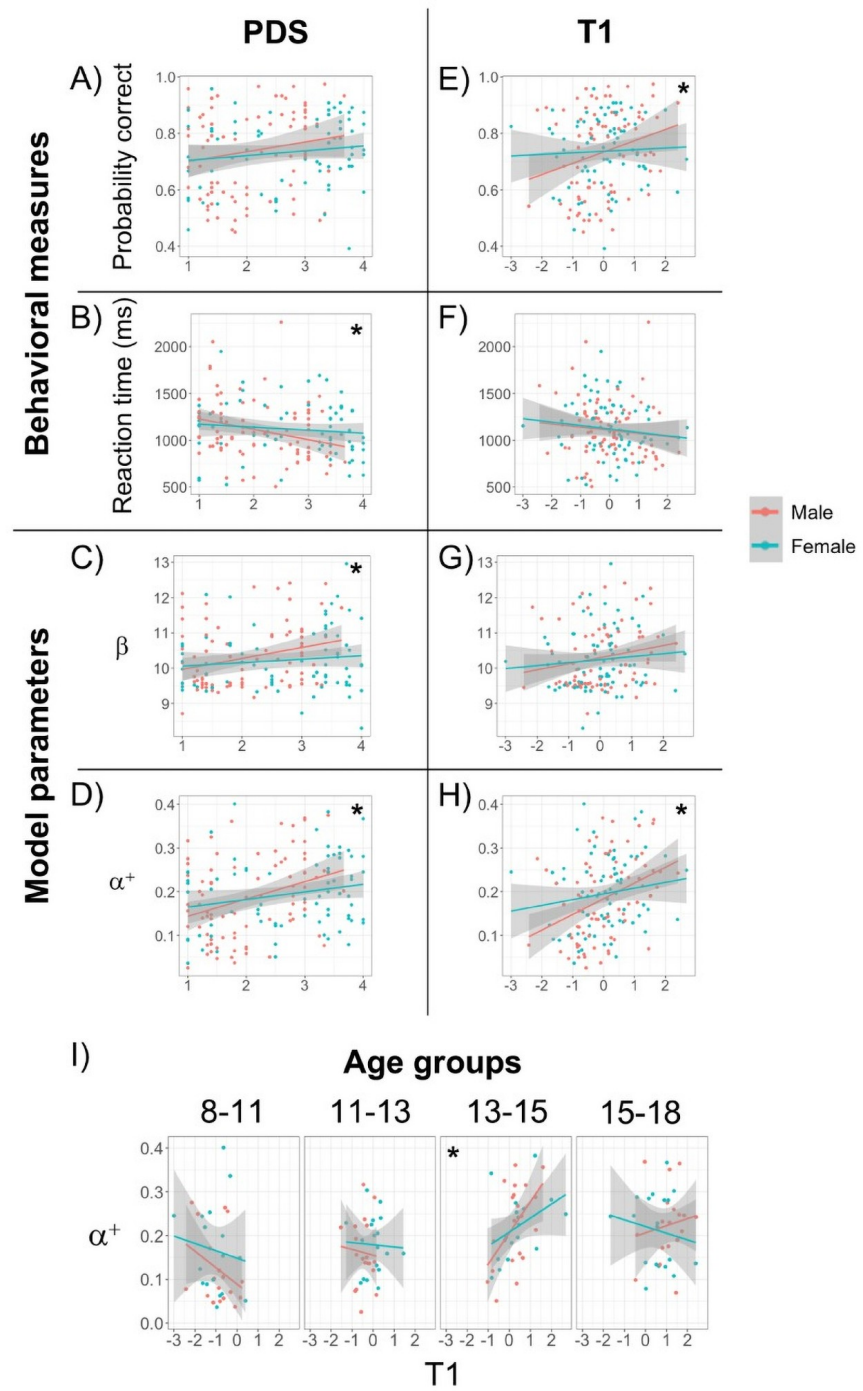
Pubertal effects on behavioral measures and model parameters. Scatter plots of (A) Puberty development scale (PDS) vs. overall performance, (B) PDS vs. median reaction time, (C) PDS vs. *α*^+^, and (D) PDS vs. *β*. (E-H) Same as (A-D) but with z-scored testosterone level (T1). (I) Scatter plot of T1 vs. *α*^+^ in each of the four age groups younger than age 18. Each colored dot represents one participant under 18. Color indicates self-reported sex. There were no significant effects of sex or interactions. The lines represent best fitting linear regression lines. Shaded region represents 95% confidence interval. * indicates a significant main effect of PDS or T1 (see main text).

To study whether pubertal development also affected participants’ learning and decision making, we used pubertal measures (pubertal development score PDS and testosterone level T1) to predict behavioral measures and fitted model parameters (Fig 5). Note that these analyses were only conducted on participants under 18.

Regarding the behavioral measures (Fig 5A, 5B, 5E and 5F), we found that T1, but not PDS, had a marginal linear effect on overall performance (linear regression. PDS: *β*_*PDS*_ = 0.1, 95% CI = [−0.04, 0.24], *p* = 0.18; T1: *β*_*T*1_ = 0.14, 95% CI = [0, 0.27], *p* = 0.05). PDS, but not T1, had a marginal linear effect on reaction time (linear regression. PDS: *β*_*PDS*_ = −0.05, 95% CI = [−0.1, 0], *p* = 0.05; T1: *β*_*T*1_ = −0.03, 95% CI = [−0.07, 0.02], *p* = 0.25). When controlling for age in the regression, the effect of T1 on performance disappeared (multiple linear regression, *p*(*T*1) = 0.53, *p*(*age*) = 0.001). However, for the effect of PDS on reaction time, adding age did not improve model fit (sequential ANOVA, *F*(1, 153) = 0.1, *p* = 0.76). Note that these results are generally consistent with our previous findings relating age and behavioral measures (Fig 3), as expected given the strong correlation between age and puberty measures (see S11 Fig), albeit noisier. There was no effect of sex in any of the regressions (multiple linear regression, all *p*’s > 0.16).

For the fitted model parameters (Fig 5C, 5D, 5G and 5H), we found that both PDS and T1 had a linear effect on *α*^+^ (linear regression. PDS: *β*_*PDS*_ = 0.02, 95% CI = [0.01, 0.04], *p* = 0.0007; T1: *β*_*T*1_ = 0.02, 95% CI = [0.01, 0.04], *p* = 0.0006), and no effect on the forgetting parameter *f* (linear regression; both *p*’s > 0.5). There was a linear effect of PDS, but not T1, on *β* (linear regression. PDS: *β*_*PDS*_ = 0.15, 95% CI = [0.003, 0.29], *p* = 0.046; T1: *β*_*T*1_ = 0.13, 95% CI = [−0.01, 0.27], *p* = 0.08). However, the effects of PDS and T1 on *α*^+^ and *β* disappeared when adding age into the regression (multiple linear regression, all *p*’s > 0.55), while age remained the only significant predictor (multiple linear regression, all *p*’s < 0.027). There was no effect of sex in any of the regressions (multiple linear regression, all *p*’s > 0.2).

To further explore the effect of PDS and T1 on model parameters while controlling for age, we performed the same regression using PDS or T1 to predict model parameters within each of the four age groups under 18 (Fig 5I and S12 Fig), within which age and puberty measures were less correlated (S4 Table). We found that within the third age group (Fig 5I) (age 13–15), there was a positive effect of T1 on *α*^+^ (linear regression: *β*_*T*1_ = 0.05, 95% CI = [0.01, 0.08], *p* = 0.005). This remained significant when correcting for multiple comparisons (two parameters by four groups). This T1 effect remained when controlling for age in the regression (multiple linear regression: *β*_*T*1_ = 0.05, *p*(*T*1) = 0.006, *p*(*age*) = 0.95), as T1 and age were decorrelated in this age group (*ρ* = −0.03, *p* = 0.85; S4 Table). T1 did not provide additional explanatory power for *β* and *f* parameters (see S1 Text: Pubertal effects extended).

## Discussion

How do humans learn to make choices when the outcome is uncertain? To learn probabilistic contingencies, humans need to integrate information over multiple trials to avoid overreacting to noise in the environment. But to learn efficiently, they also need to pay attention to recent information. Here, we investigated how humans trade off these constraints across development, what the underlying computational mechanisms that support such learning are, and how they change during adolescence.

At the population level, computational model comparison (Fig 2B) suggested that two mechanisms modulated learning of probabilistic contingencies. First, participants did not treat positive and negative feedback identically; rather, they had a strong bias to learn more from positive, and little to none from negative feedback. This asymmetry has been widely observed in previous studies [12, 24, 31], potentially due to differential mechanisms integrating positive and negative feedback [48]. Second, we found that learning was better explained by including a forgetting mechanism: more intervening trials between two iterations of a choice decreased the strength of past information [12].

Consistent with the age effects observed in previous work using tasks with probabilistic [3] and deterministic [12] feedback, our behavioral and modeling results suggest that learning in a stable probabilistic task environment changed markedly from childhood to adulthood. In particular, we found that overall performance increased with age, stabilising in early adulthood. This behavioral pattern was mirrored by the learning rate parameter (*α*^+^) as well as inverse temperature (*β*), a parameter indicating a decrease in noise or exploration in choice.

Our observations that learning rate *α*^+^ and inverse temperature *β* increase with age and pubertal development during adolescence are generally consistent with previous work using the deterministic learning task *RLWM*, tested in the same participants as shown here [12], and a probabilistic task with same the same overall task structure as the Butterfly task, but different feedback methods [2]. However, we did not find higher performance in adolescents than adults, as had been observed in this previous Butterfly task study [2] (Fig 3A). Even when using the same age bins as [2], which limited our number of participants to N = 84 13–18-year-old adolescents and N = 86 20–30-year-old adults, we instead found that the performance in 20–30-year-olds was significantly higher than 13–18-year-olds (unpaired t-test, *t*(168) = 2.3, *p* = 0.02). Because our 18–25 year-olds were differently recruited than the rest of our sample, we additionally compared the 13–18-year-old adolescents to N = 54 25–30-year-old adults (i.e. not including the undergraduate participants between 20–25 years old). We still found significantly better performance in adults (unpaired t-test, *t*(136) = 2.2, *p* = 0.03).

The finding in [2] was interpreted as “an upside” to slower learning that led to more robust integration over time of information, and thus higher overall performance under uncertainty at younger ages. Indeed, lower learning rates can be more optimal in probabilistic tasks than higher learning rates. However, the relationship between learning rates and performance when learning probabilistic contingencies is complex and non-monotonic: it follows an inverse U-shape, as very low learning rates lead to integrating information too slowly, but high learning rates lead to being too susceptible to noisy feedback (see S1 Text: Nonlinear relationship between performance and model parameters, [44]). Furthermore, the inverse U-shape itself is dependent on the degree of exploration and forgetting ([2, 4, 44], see supplementary simulations in S7 and S8 Figs). Learning rates were smaller in our study compared to [2]: the group level mean for *α*^+^ in our sample was 0.18, whereas in [2], the mean was around 0.3 and 0.6 for adolescents and adults respectively (Fig 2B in [2]). In higher ranges of learning rates [2], an increase in learning rate could result in a decrease in performance (right side of the inverse U-shape), while in our lower range, it could lead to an increase in performance (left side of the inverse U-shape). Thus, the two studies are consistent in identifying an increase in learning rate with age, but over a different range of learning rate values (0.3 vs. 0.6), leading to opposite effects on performance. Indeed, in our study, the parameter trajectory with age corresponded to a slow improvement towards more “optimal” behavior, as defined by correct performance in the task (S7 Fig).

Moreover, we modeled learning from positive and negative feedback asymmetrically [4], as opposed to the symmetric learning rate in [2]. In particular, our winning model *α*^+^0*βf* did not learn from negative feedback at all. A high *α*^−^ can also result in worse asymptotic performance in the Butterfly task (see S9 Fig), resulting in more switching from the preferred flower. Note that when using the same model as in [2] with symmetric learning rate, i.e. the *αβ* model, we found similar age effects (see S1 Text: Age effects in the *αβ* model) on the *α* and *β* parameters as on the *α*^+^ and *β* parameters in the winning model *α*^+^0*βf*.

Therefore, while we found a similar trend as in [2] that learning rates increased with age (Fig 4A), our learning rate values were much smaller, and the resulting trend in overall performance was different. Note that this difference in the range of learning rates could be a result of differences in the task specifics (our experiment did not have a memory retrieval aspect with novel images or brain imaging; our task was also the third in a sequence of four tasks). Differences in performance could also stem from differences in socioeconomic status (SES) and education level between the groups recruited to each study. For example, our 18–25 year-olds were undergraduate students, who may have a different education level than the 25–30 year-old community participants in our study or the adults sampled in [2].

The incentivization for undergraduate participants (course credit) and community participants (monetary) was also different. Furthermore, participants’ performance (total points earned) was not translated into real-life reward such as money. While there have been studies showing that the human brain treats primary and secondary reinforcers similarly [49–52], the importance of incentivization remains a controversial topic in decision making research. It is thus unclear whether there are developmental differences regarding how the incentive structure in the Butterfly task might motivate participants differently at different ages.

Another potential limitation of our sample is that the majority of the excluded participants were under 18, which could contain meaningful variance. This might be due to the fact that younger participants were a bit more likely to be distracted during the task/had a more difficult time understanding the task logistics, although most (about 90%) of our younger participants were able to understand and engage well in the task to pass this criterion. In particular, we excluded participants who, for a given butterfly, were more likely to switch than stay after positive feedback (see S1 Text: Exclusion criteria details), because these participants showed no reward sensitivity and were thus likely “off-task”. Since we focused on developmental changes in learning, we found it necessary and helpful for later modeling analysis to exclude participants by this criterion. The parameters from those participants would not be interpretable since the RL models all assume trial-by-trial learning from reward feedback, which these participants’ behavior demonstrated a lack of. Moreover, even if we included them in modeling, they would likely have very low learning rates (since they are not sensitive to positive feedback) and low *β* (random/noisy behavior), which could only strengthen our findings. Finally, since the study is cross-sectional rather than longitudinal, age could also be confounded by birth year.

Nevertheless, our results support other previous developmental findings. In particular, we also found a decrease in exploration with age [1, 12], and an increase in learning rate previously observed in both deterministic [12] and probabilistic learning tasks [3]. Note that other studies have observed a decrease in learning rates (e.g., single-learning-rate models: [25, 53, 54]; models with asymmetric learning rates: [24, 31, 55]) or no change [36, 37]. These differences are potentially due to different task structures, samples, and modeling choices. For a more comprehensive review, see [4]).

While we found that performance increased during adolescence and stabilized in early adulthood in this stable probabilistic learning task, a probabilistic switching task in the same sample of participants [3] found a pronounced inverse U shape in overall performance, which peaked at age 13–15. We conclude that this difference in age of peak performance in these two tasks stems from the reliance or lack of reliance on negative outcomes. The stable associations in the Butterfly task might encourage the participants to focus mostly on positive feedback (although this is not optimal based on the simulations in S9 Fig, as *α*^−^ in the low range can improve performance), whereas in a volatile task setting [3], negative feedback was crucial for identifying when the correct action switched. This suggests that even with the same sample of participants in two probabilistic tasks, task stability / volatility greatly changed participants’ behavioral strategies. For this sample of participants, the volatile condition in [3] gave the 13–15 year old adolescents an edge over adults, while the stable condition in the Butterfly Task gave young adults an edge over adolescents.

While we found that random effects of delay on performance (calculated from the mixed effect logistic regression, which is descriptive) became more pronounced with age (Fig 3D), computational model fitting in contrast showed that forgetting parameters became weaker (Fig 4C). One possible interpretation for this apparent contradiction might relate to two simultaneous changes. First, adults might rely more on working memory processes [19] for probabilistic tasks [56], which manifested in a strengthened effect of delay. However, the decay of these memory processes might also decrease with age [12], which could be captured here by the decrease in the forgetting parameter. Thus, younger participants might show a weaker effect of delay not because their memory system was forgetting less (it was forgetting more), but because they used their working memory system less in this task [12, 57], and instead relied more on slower but more robust learning systems.

While we found that pubertal measures did not explain much additional variance compared to age in model parameters (see S1 Text: Pubertal effects extended), we found that testosterone level T1 had a significant positive effect on *α*^+^ within the third age group of 13–15 years (Fig 5I). Several explanations for this time limited observation are possible: a) gonadal hormone effects are stronger at this time of mid puberty, b) the other individual drivers of variation are more consistent at this time allowing detection of puberty related effects, or c) this result is a type I error. We favor hypothesis a) and b) because this observation about learning rate is broadly consistent with several studies [7, 29, 30] which report a positive relationship between testosterone levels and nucleus accumbens bold activity in response to rewards in mid adolescence. These data combined suggest a putative link between testosterone, nucleus accumbens activity, and learning rate in mid adolescence. Our testosterone related findings may also be relevant to a putative sensitive period for social learning driven by gonadal hormones in adolescence [13, 58]. Future experiments may test if these relationships between learning rate and testosterone in this non-social context are replicated and/or magnified in a social context.

Overall, work on the role of puberty and learning is currently in an early phase of understanding. It is likely that there are gonadal hormone dependent and independent aspects of development in the brain that will need to be disentangled [59]. A longitudinal design will have stronger statistical power to isolate puberty dependent effects [11]. Other sources of hormones and neuropeptides may also contribute to coordinate developmental change across the body and cumulative experience may also contribute. Age is less noisy to measure than pubertal development or hormones, but age is not a satisfactory explanation at the proximate level of analysis which aims to identify upstream biological mechanisms.

## Conclusion

In conclusion, we sought to examine the development of learning in a stable probabilistic environment using a large adolescent and young adult sample with continuous age in the 8–30 range. Combining behavioral analysis and computational modeling, we showed developmental gains in performance through early adulthood that were explained by an increase in learning from rewarded outcomes (corresponding to a narrower time scale of information integration) and a decrease in exploration. These data and models help explain why learning and decision making differ during development and why a ‘one-size-fits-all’ approach may not equally serve youth at different stages.

## Supporting information

S1 Text Supplementary results. (PDF)

S1 Fig Demographics. (TIF)

S2 Fig Generate and recover. (TIF)

S3 Fig Successful generate and recover of *α*^−^ in a higher and healthier range. (TIF)

S4 Fig Model validation. (TIF)

S5 Fig Generate and recover for the age regression coefficients in hierarchical modeling. (TIF)

S6 Fig Flat model comparison of all six models per age group. We calculated the difference between the BIC for each of the six models with *α*^+^0*βf* model per participant, represented by ΔBIC on the y-axis. Color represents 6 age groups. Results show that the *α*^+^0*βf* model is the winning model in all groups consistently.
(TIF)

S7 Fig Heat map for simulated performance of the *α*^+^0*βf* model. Overall simulated performance changes with respect to *α*^+^ (y-axis) and *β* (x-axis), where each subplot corresponds to *f* = 0 − 0.2 from left to right. Black rectangle highlights the local maximum within each column of each subplot (i.e. fixed *β* value), whereas the red rectangle highlights the global maximum.
(TIF)

S8 Fig Heat map for simulated performance of the *α*^+^0*βf* model. Overall simulated performance changes with respect to *α*^+^ (y-axis) and *f* (x-axis), where each subplot corresponds to *β* = 5 − 15 from left to right. Black rectangle highlights the local maximum within each column of each subplot (i.e. fixed *f* value), whereas the red rectangle highlights the global maximum.
(TIF)

S9 Fig Simulated performance of the *α*^+^*α*^−^*β* model. Overall simulated performance (y-axis) changes with respect to *α*^−^ (x-axis), where each subplot corresponds to a combination of (*α*^+^, *β*) values. The vertical bar corresponds to the *α*^+^ value. The error bars show standard error across 100 simulations.
(TIF)

S10 Fig Age effects on the *αβ* model parameters. We directly incorporated age-related parameters into MCMC sampling to test within the hierarchical Bayesian modeling framework whether age had a linear or quadratic effect on the fitted parameters from the *αβ* model. We found positive linear effect of age on *α* and *β*, and negative quadratic effect of age on *β*. The model with quadratic age effect on *α* failed to converge.
(TIF)

S11 Fig Scatter plots of pubertal measures (PDS and T1) and age. (TIF)

S12 Fig Pubertal effects on behavior and fitted *α*^+^ parameters in each of the four age groups younger than age 18. (A) PDS vs. median reaction time. (B) T1 vs. overall performance. (C) PDS vs. *α*^+^. (D) T1 vs. *α*^+^.
(TIF)

S1 Table Number of participants excluded due to each exclusion criterion for each of the four age groups. (TIF)

S2 Table Age boundaries for each of the four age groups under 18. (TIF)

S3 Table Model identifiability analysis. The rows indicate the model where the dataset was generated from, whereas the columns indicate the model used for recovery. Each entry indicates protected exceedance probability.
(TIF)

S4 Table Statistics of pubertal measures under 18. Within each of the age group under 18, we calculated the variance of pubertal measures (PDS and T1) and their correlations to age.
(TIF)
