# Supplementary material for: Modeling changes in probabilistic reinforcement learning during adolescence
Source: PLoS Comput Biol. 2021 Jul 1;17(7):e1008524. doi: 10.1371/journal.pcbi.1008524 (PMC8279421; doi:10.1371/journal.pcbi.1008524)
Supplement: S1 Text — (PDF) [file pcbi.1008524.s001.pdf]

S1 Text: Supplementary Text for Modeling Changes in Probabilistic Reinforcement Learning during Adolescence

Liyu Xia, Sarah L Master, Maria K Eckstein, Beth Baribault, Ronald E Dahl, Linda Wilbrecht, Anne Gabrielle Eva Collins

Exclusion criteria details

We collected data from 297 participants on the Butterfly task, with one participant excluded for only having 18 trials of data. We then excluded 21 participants who were more likely to switch than stay after positive feedback.

So far 275 participants remained. To further identify participants who were not engaged in the task without excluding participants solely on a pure performance criterion, we instead implemented the following, less stringent, *conjunctive* exclusion criteria:

- Criterion 1. Proportion of stay trials (the participant picked the same flower as the previous trial regardless of the butterfly) was higher than $median + 2 * sd$, across the whole group.
- Criterion 2. Proportion of stay trials was lower than $median - 2 * sd$, across the whole group.
- Criterion 3. The number of contiguous stay trials was higher than 12 contiguous stay trials in a row.
- Criterion 4. Any number of missing trials: available data less than the full 120 trials, indicating that participants stopped before the end of the experiment.
- Criterion 5. Performance was not better than chance (50%): performance was based on proportion of trials where participants correctly chose the preferred flower of the butterfly.

We excluded participants who fit **both** Criterion 5 and one of Criterion 1-4. Criteria 1-3 revealed participants who were either choosing the same action (e.g. always choosing left) or constantly switching between the two actions without a relationship to the butterfly on the screen or the task at all. Together, Criteria 1-4 are likely to include participants who either did not understand the instructions or were not engaged for a significant proportion of the task. Finally, we

took the intersection with Criterion 5 (at or below chance performance), so that we only excluded participants whose lack of understanding or disengagement (one of Criteria 1-4) significantly impacted their performance to be at or below chance (Criterion 5). Note that there were actually many participants who had worse than chance performance (Criterion 5), but were not excluded because they did not meet any of Criteria 1-4, which were indicators of not paying attention/misunderstanding the task. S1 Table shows the detail breakdown of participants' age group for each criterion.

After applying this conjunctive criteria, we further eliminated 11 participants for later analysis. In the end, we analyzed $N = 264$ participants, with 157 participants younger than 18. S1 Fig shows more detailed age group and sex break down. As mentioned in the main text, all results remain qualitatively similar with weaker exclusion criteria.

The exact age boundaries for the four age groups that we defined for participants under 18 are summarized in S2 Table.

Model comparison extended

Although the $\alpha^{+}\alpha^{-}\beta f$ model has better model comparison score, our analysis focuses on the simpler $\alpha^{+}0\beta f$ model as the winning model because (1) α^{-} was not recoverable and (2) $\alpha^{+}0\beta f$ validated participants' behavior better. This is in following with a large literature indicating that quantitative criteria for model comparison are not the single factor that should guide model selection [7, 12, 13, 9].

For fitted parameters, it is important to first verify if we can recover them from simulated data [10]. Specifically, because RL models are generative, we can simulate artificial data from fitted parameters and fit the model on the artificial data again to check the identifiability of the model parameters and the robustness of the model fitting procedure.

For the $\alpha^{+}\alpha^{-}\beta f$ model, we were unable to recover the α^{-} parameter (S2A Fig). For the $\alpha^{+}0\beta f$ model, all three parameters could be reasonably recovered (S2B-D Fig).

Note that this poor recoverability of α^{-} might be primarily due to its small values. To test this hypothesis, we fitted the $\alpha^{+}\alpha^{-}\beta f$ model to participants data, and then fixed the α^{+}, β and f parameters, but substituted new values for the α^{-} parameter, sampled from a broader range (Gaussian $N(0.5, 0.2)$ truncated $T[0.1, 0.9]$). We then performed the generate and recover analysis with this set of parameters. As shown in S3 Fig, the α^{-} values could be well recovered in this higher range of values.

We further validated the fitted models by visualizing the learning curves of simulated data and comparing with participants' data (S4 Fig). Note that since the $\alpha\beta f$ model did not converge hierarchically (see Hierarchical model fitting), the simulation for the $\alpha\beta f$ model came from fitting each participant independently (i.e. flat instead of hierarchical fitting), while all the other five model simulations came from hierarchically fitted parameters.

Overall, the $\alpha^{+}0\beta$ and $\alpha^{+}0\beta f$ models both track participants' learning curve well throughout the experiment, whereas all the other models overshoot to various extent. This combined with the fact that α^{-} was not recoverable suggests that the $\alpha^{+}\alpha^{-}\beta f$ model, despite having a better model comparison score (Fig 2B), had a risk of overfitting.

Since the values of the forgetting parameter are fairly small, it is not surprising that the simulations of the $\alpha^{+}0\beta$ and $\alpha^{+}0\beta f$ models do not differ a lot. We also note that it is possible that the forgetting parameter captures patterns of data not readily visible in the learning curve, since delays between same butterflies are randomized across participants.

We also performed generate and recover procedure to validate the significant age regression coefficients from hierarchical modeling (i.e. linear and quadratic age effects on α^{+} and β; Fig 4). S5 Fig shows that the posterior of the samples from the recovery matched the posterior of the samples fitted on actual participants data fairly well for all significant age regressors.

While the generate and recover analysis so far shows good recoverability of fitted parameters in our winning model $\alpha^{=}0\beta f$, we further validated model identifiability/recoverability, i.e. we asked the question of when we generate data from, say, Model A, whether Model A would still be the winning model among others for explaining this data.

Since we fitted all participants jointly, the ideal analysis would be to simulate from the fitted parameters and use all models to fit this simulated dataset hierarchically and report WAIC. Moreover, this should be performed multiple times [8]. However, each hierarchical fitting takes 10 hours to complete, resulting in significant computational overhead.

Instead, we decided to reduce the amount of compute necessary by fitting participants independently. To further reduce compute, we focused only on the $\alpha\beta$, $\alpha^{+}0\beta$, and $\alpha^{+}0\beta f$ models, since our model comparison results mostly rely the identifiability of (1) asymmetric learning rates with $\alpha^{-} = 0$ and (2) the forgetting parameter. We simulated a dataset from each of the three fitted flat models and used all three models to fit these three datasets, again non-hierarchically. For the data simulated from each model, we calculated the BIC for each of the three models used for recovery and each participant separately, and used BIC as an approximation of model evidence [6] to calculate protected exceedance probabilities [1, 4]). The exceedance probabilities are summarized in the following confusion matrix (S3 Table), showing great model identifiability.

We also compared all six models when fitted non-hierarchically (S6 Fig). We used the winning model in the main text, $\alpha^{+}0\beta f$, as the baseline to calculate the difference between the BIC of all other models with the $\alpha^{+}0\beta f$ model. S6 Fig shows that the $\alpha^{+}0\beta f$ model is a decent model in all age groups consistently.

Nonlinear relationship between performance and model parameters

The model's overall performance in this task depends nonlinearly on the interaction of all parameters. To better illustrate this nonlinear relationship, we simulated the $\alpha + \alpha - \beta f$ model with the following parameter values: α^+ (0.01, 0.1, 0.2, 0.3, 0.4, 0.5, 0.6, 0.7), α^- (0, 0.1, 0.2, 0.3, 0.4, 0.5, 0.6, 0.7), β (5, 7.5, 10, 12.5, 15), and f (0, 0.05, 0.1, 0.15, 0.2). Each parameter combination is simulated 100 times.

For simplicity of visualization, we first considered the winning model $\alpha^+ 0 \beta f$, i.e. by setting $\alpha^- = 0$, and focus on the discrete grouping of participants. The following heat maps (S7 Fig) show overall simulated performance change with respect to α^+ (y-axis) and β (x-axis) for different forgetting values. Indeed, as mentioned in the discussion, increasing learning rate α^+ does not always improve performance; in fact, too high of a learning rate can result in suboptimal behavior ([10]). All six age groups (four under 18, two above 18) had average β values around 10 (from young to old: 10.12, 9.95, 10.54, 10.46, 10.85, 10.99) and average f values around 0.05 (0.0669, 0.0555, 0.054, 0.0553, 0.0554, 0.0545), roughly corresponding to the second subplot's third column (S7 Fig). The average α^+ values for each age group are 0.14, 0.17, 0.22, 0.22, 0.26, 0.25 (plotted on the heat map as colored circles, darker means older age group), where higher α^+ generally improves overall performance. The simulations thus show that all parameter changes with age in our sample go towards more "optimal" performance in this task, but remain fairly far from it.

Another interesting observation is that no forgetting is not always optimal, especially in scenarios where the learning rate is high. This is more visible in S8 Fig, where we interchange the role of β and f from the previous heat map (now each subplot is indexed by β values from 5 - 15 and the x-axis of each subplot represents forgetting). This might be due to the fact that forgetting can actually help unlearn the suboptimal win-stay/lose-shift behavior created by high learning rates and/or highly exploitative policies.

In general, within the range of 5 - 15 for β, overall performance increases with higher β. And the optimal α^+ seems to be inversely related to β but positively related to f.

Similar observations to α^+ can be made for α^-. In S9 Fig, we set $f = 0$ for simplicity. Each subplot corresponds to one specific combination of (α^+, β) values, where the x-axis indicates α^- values and the y-axis overall performance. We also add a vertical bar for the corresponding α^+ value. For all combinations, while increasing α^- helps performance in the low range, high α^- always hurts performance. It is notable that optimal performance is rarely obtained with symmetric values of learning rates (i.e. the peak of the curve does not match the vertical bar). Note that since [5] employed symmetric learning rates, adult participants with high α^+ would have the same high α^- in their sample, which could also hurt performance.

Age effects in the $\alpha\beta$ model

In order to match the model used in [5], we also probed the age effect on model parameters for the $\alpha\beta$ model (see S10 Fig). We used the same hierarchical modeling technique detailed in main text (see Hierarchical model fitting). Aside from the model with quadratic age effect on α, which did not converge, all the other models successfully converged. We found a positive linear age effect on both alpha and beta (95% CI, $\alpha : [0.04, 0.13], \beta : [2.45, 6.19]$), as well as a negative quadratic age effect on beta (95% CI = $[-5.77, -0.44]$), all corroborating developmental results regarding the $\alpha^+0\beta f$ model (Fig 4A and 4B).

However, we would also like to caution that the $\alpha\beta$ model does not capture participants' behavior in our study well (see Figs 2 and S4A). This is potentially due to the fact that the α^- parameter is very small (0 in the winning model $\alpha^+0\beta f$), and thus when fitting models with symmetric learning rates (i.e. setting $\alpha^+ = \alpha^-$), α^- drags down the α^+ parameter as well, making the final learning rate parameter α very small (group level mean for α in the $\alpha\beta$ model is 0.02). Since the $\alpha\beta$ model does not capture behavior well, using it as a direct comparison with [5] is not ideal. Therefore, in the main text (see Discussion) we focused on the comparison of the α^+ and α^- (set to 0) parameters in the $\alpha^+0\beta f$ model (the winning model) to the α parameter in [5] respectively, which still contains the same amount of information from both studies.

Saliva collection and testosterone testing

In addition to self-report measures of pubertal development, we also collected saliva from each of our participants to quantify salivary testosterone, following methods reported in [14]. Testosterone is a reliable measure of pubertal status in boys and girls and is associated with changes in brain and cognition in adolescence [3, 2]. Participants refrained from eating, drinking, or chewing anything at least an hour before saliva collection. Participants were asked to rinse their mouth out with water approximately 15 minutes into the session. At least an hour into the testing session, they were asked to provide 1.8 mL of saliva through a plastic straw into a 2 mL tube. Participants were instructed to limit air bubbles in the sample by passively drooling into the tube, not spitting. Participants were allotted 15 minutes to provide the sample. After the participants provided 1.8 mL of saliva, or 15 minutes had passed, the sample was immediately stored in a -20°F freezer. The date and time were noted by the experimenter. The participants then filled out a questionnaire of information which might affect the hormone concentrations measured in the sample (i.e. whether the participants had recently exercised).

Salivary testosterone was quantified using the Salimetrics Salivatory Testosterone ELISA (cat. no. 1-2402, Bethesda, MA). Intra- and inter- assay variability for testosterone were 3.9% and 3.4%, respectively. Samples below the detectable range of the assay were assigned a value of 5 pg/mL, 1 pg below the lowest detectable value. Final testosterone sample concentration data were

cleaned with a method developed in [11]. There were no participants with any samples above the detectable range, i.e. the measured values of the testosterone concentration for all participants were within the range that we validated as detectable by our technique. Within participants aged 8 to 17 only, outliers greater than three standard deviations above the group mean were fixed to that value, then incremented in values of +0.01 to retain the ordinality of the outliers. For T1 used in the manuscript, we log-transformed raw testosterone levels, which otherwise was more skewed and did not pass normality tests.

We visualized the relationships between pubertal measures and age for our sample of participants in S11 Fig.

Pubertal effects extended

Here we aggregate all results for pubertal effects on behavioral measures and model parameters for participants under age 18. To control for age, participants were broken into four equal-sized groups within each sex respectively and then combined. We also included a table of the variance of the pubertal measures and the correlation between the pubertal measures with age within each of the four age groups (see S4 Table).

To better explore the effect of PDS and T1 on behavioral measures while controlling for age, we performed the same regressions as in Fig 5 using PDS or T1 to predict overall performance and reaction time within each of the four age groups under 18 (see S12A and S12B Fig). We found that in the fourth age group (age 15 - 18), PDS had a linear effect on reaction time ($\beta_{PDS} = -0.2$, 95% CI = $[-0.36, -0.03], p = 0.02$), which became marginal when correcting for multiple comparisons (four groups, $p = 0.08$). This PDS effect remained when controlling for age in the regression (multiple linear regression: $\beta_{PDS} = -0.2, p(T1) = 0.02$, $p(age) = 0.6$). T1 did not provide additional explanatory power for overall performance in any of the four age groups under 18 (all p's > 0.26). These effects would not survive multiple comparison correction, and as such should be viewed as mostly exploratory.

To test pubertal effects on model parameters in addition to age, within each age bin, we used either PDS or T1 to predict model parameters with linear regression. After controlling for age (S12C Fig), we did not find significant effects of PDS on α^+ in any of the four age bins (linear regression, all p's > 0.37). We did find a significant effect of T1 on α^+ (S12D Fig) in the third age bin (linear regression, $\beta_{T1} = 0.0008, p = 0.005$), but not in the other three age bins (linear regression, all p's > 0.17).

References

[1] Klaas Enno Stephan et al. “Bayesian model selection for group studies”. In: *Neuroimage* 46.4 (2009), pp. 1004–1017.

[2] JS Peper et al. “Sex steroids and brain structure in pubertal boys and girls: a mini-review of neuroimaging studies”. In: *Neuroscience* 191 (2011), pp. 28–37.

[3] Megan M Herting et al. “The role of testosterone and estradiol in brain volume changes across adolescence: a longitudinal structural MRI study”. In: *Human brain mapping* 35.11 (2014), pp. 5633–5645.

[4] Lionel Rigoux et al. “Bayesian model selection for group studies—revisited”. In: *Neuroimage* 84 (2014), pp. 971–985.

[5] Juliet Y. Davidow et al. “An Upside to Reward Sensitivity: The Hippocampus Supports Enhanced Reinforcement Learning in Adolescence”. en. In: *Neuron* 92.1 (Oct. 2016), pp. 93–99. ISSN: 0896-6273. DOI: 10.1016/j.neuron.2016.08.031.

[6] Germain Lefebvre et al. “Behavioural and neural characterization of optimistic reinforcement learning”. In: *Nature Human Behaviour* 1.4 (2017), pp. 1–9.

[7] Stefano Palminteri, Valentin Wyart, and Etienne Koechlin. “The Importance of Falsification in Computational Cognitive Modeling”. en. In: *Trends in Cognitive Sciences* 21.6 (June 2017), pp. 425–433. ISSN: 1364-6613. DOI: 10.1016/j.tics.2017.03.011.

[8] Camile MC Correa et al. “How the level of reward awareness changes the computational and electrophysiological signatures of reinforcement learning”. In: *Journal of Neuroscience* 38.48 (2018), pp. 10338–10348.

[9] Danielle J Navarro. “Between the devil and the deep blue sea: Tensions between scientific judgement and statistical model selection”. In: *Computational Brain & Behavior* 2.1 (2019), pp. 28–34.

[10] Robert C Wilson and Anne GE Collins. “Ten simple rules for the computational modeling of behavioral data”. In: *Elife* 8 (2019), e49547.

[11] Marjolein EA Barendse et al. “Study Protocol: Transitions in Adolescent Girls (TAG)”. In: *Frontiers in psychiatry* 10 (2020), p. 1018.

[12] Gunnar Blohm, Konrad P Kording, and Paul R Schrater. “A how-to-model guide for Neuroscience”. In: *Eneuro* 7.1 (2020).

[13] Konrad P Kording et al. “Appreciating the variety of goals in computational neuroscience”. In: *arXiv preprint arXiv:2002.03211* (2020).

[14] Sarah L. Master et al. “Distentangling the systems contributing to changes in learning during adolescence”. en. In: *Developmental Cognitive Neuroscience* 41 (Feb. 2020), p. 100732. ISSN: 1878-9293. DOI: 10.1016/j.dcn.2019.100732.
