# Supplementary figures and images for: Modeling changes in probabilistic reinforcement learning during adolescence
Source: PLoS Comput Biol. 2021 Jul 1;17(7):e1008524. doi: 10.1371/journal.pcbi.1008524 (PMC8279421; doi:10.1371/journal.pcbi.1008524)

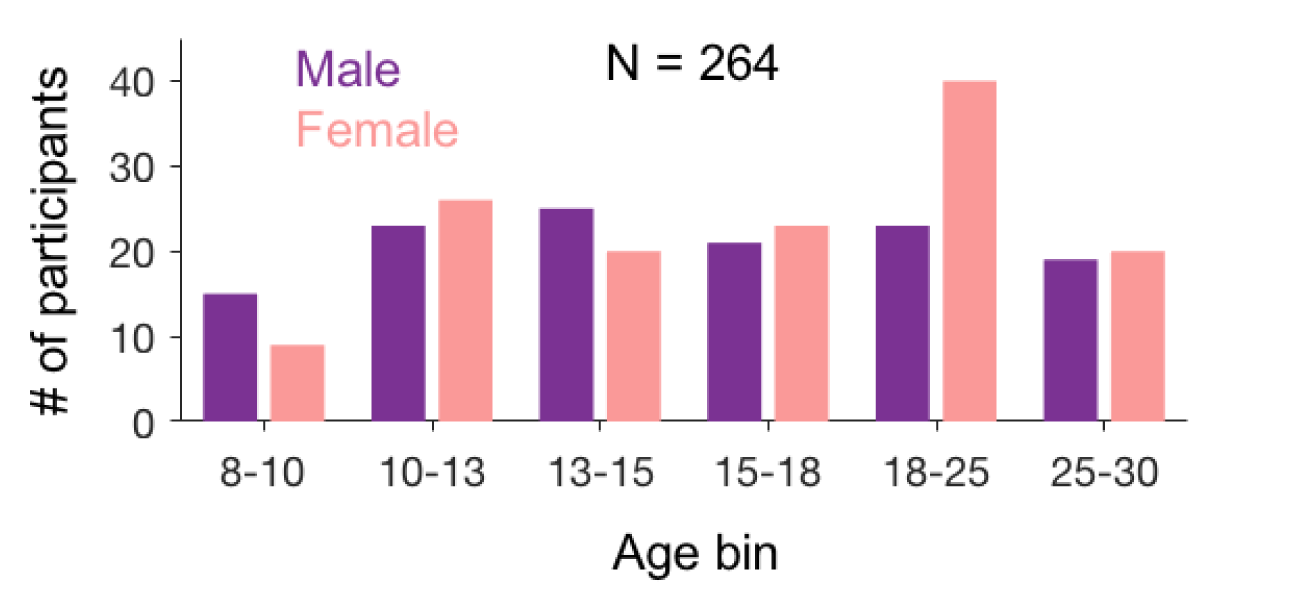

Supplement: S1 Fig — (TIF) [file pcbi.1008524.s002.tif]

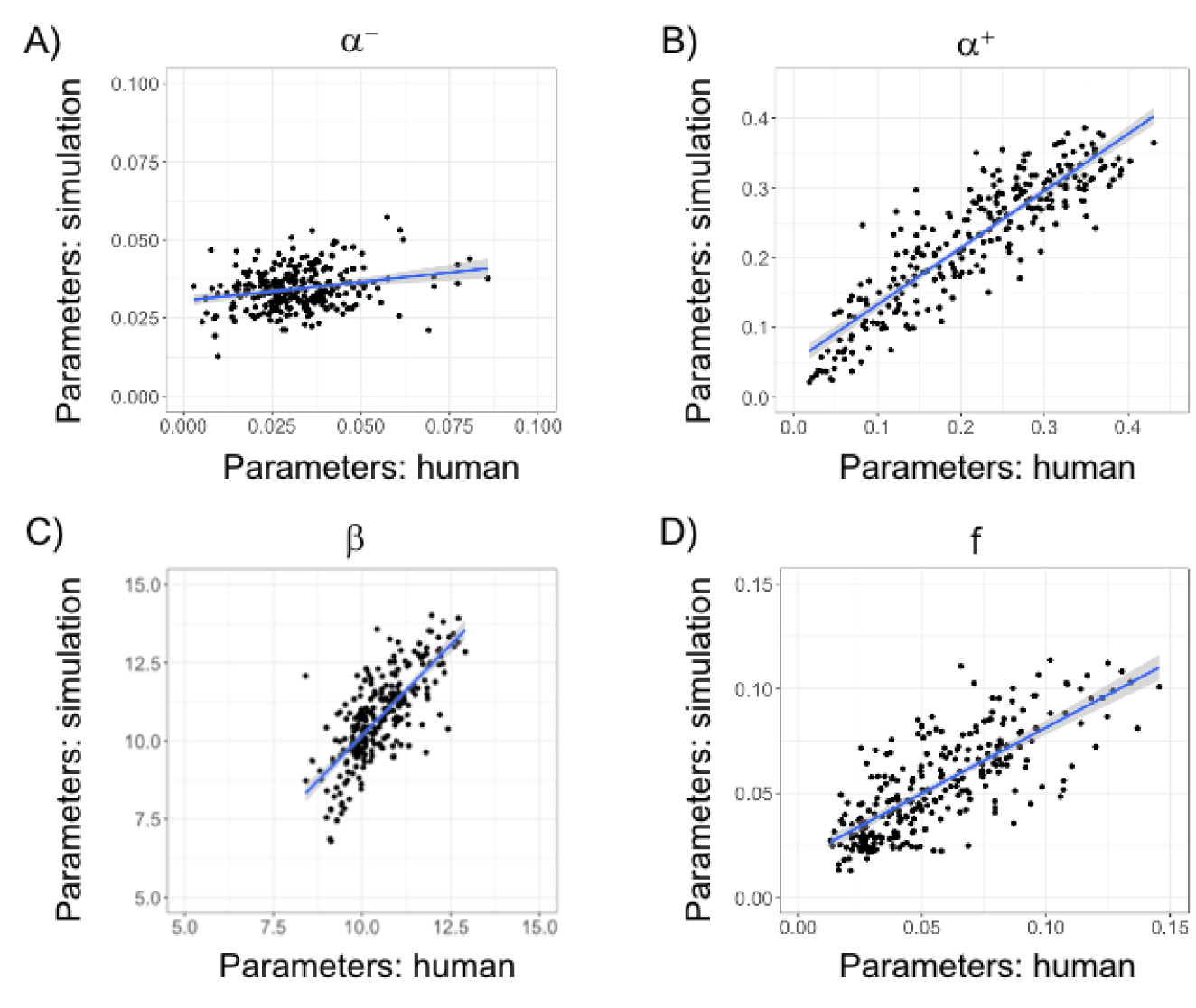

Supplement: S2 Fig — (TIF) [file pcbi.1008524.s003.tif]

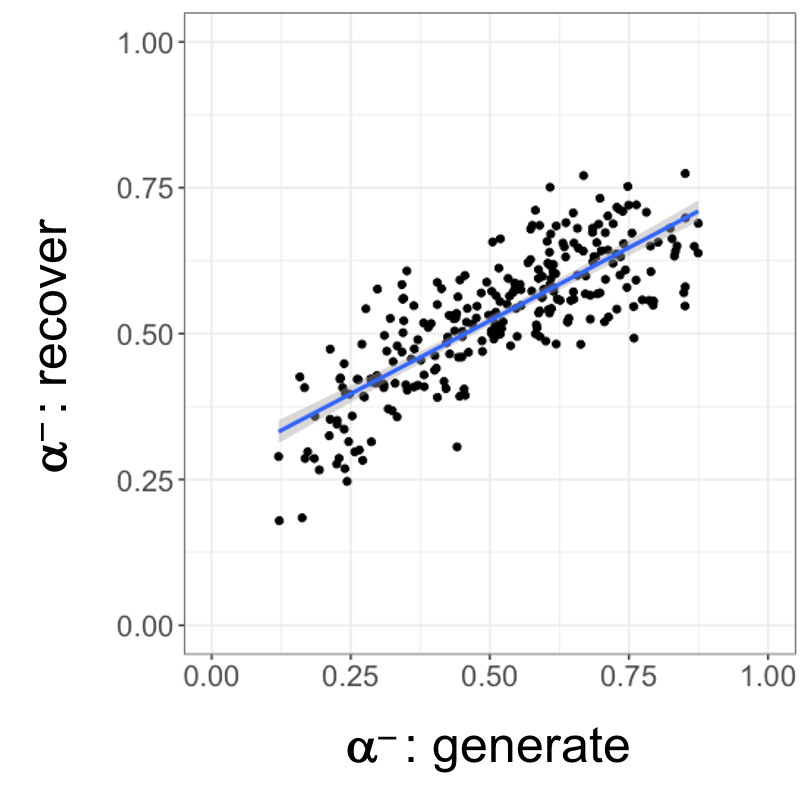

Supplement: S3 Fig — (TIF) [file pcbi.1008524.s004.tif]

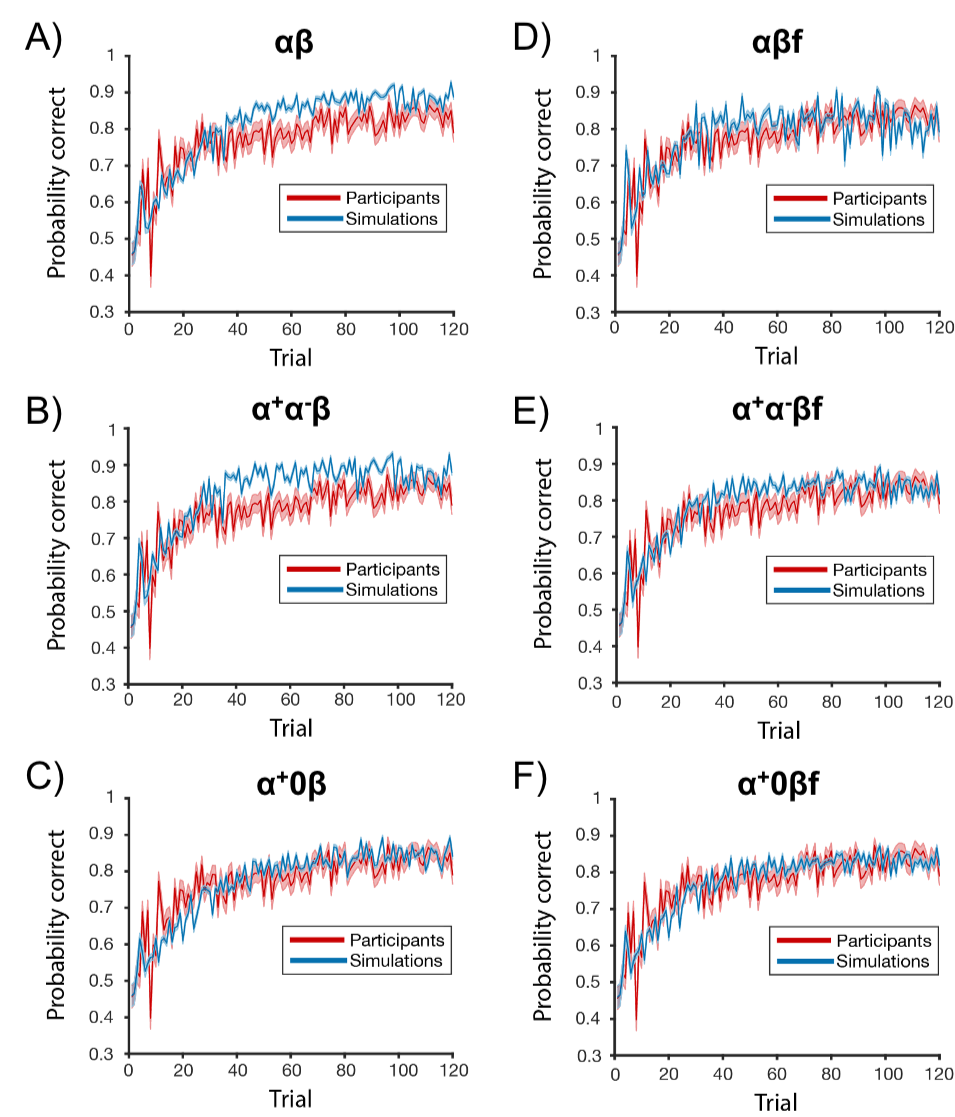

Supplement: S4 Fig — (TIF) [file pcbi.1008524.s005.tif]

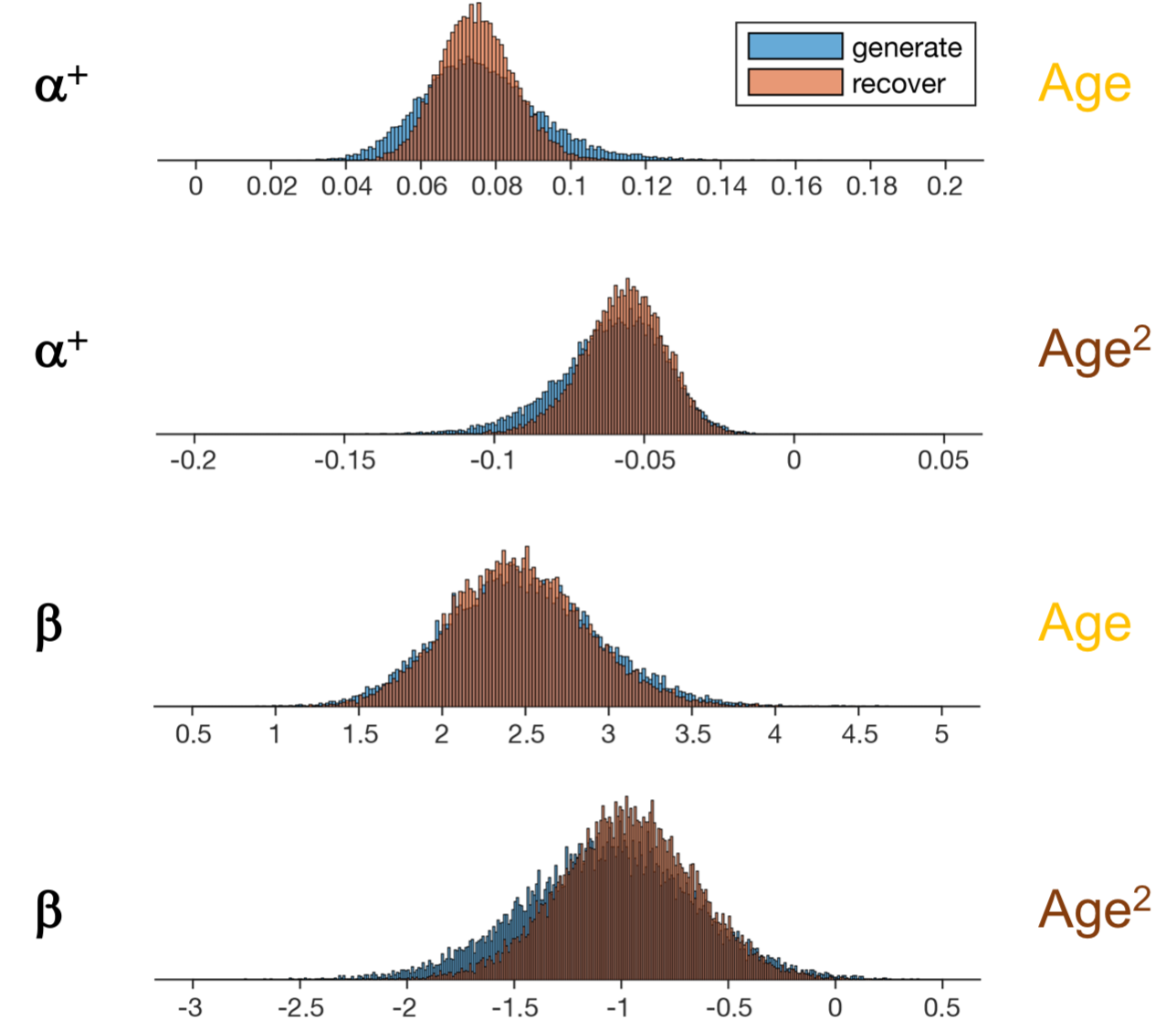

Supplement: S5 Fig — (TIF) [file pcbi.1008524.s006.tif]

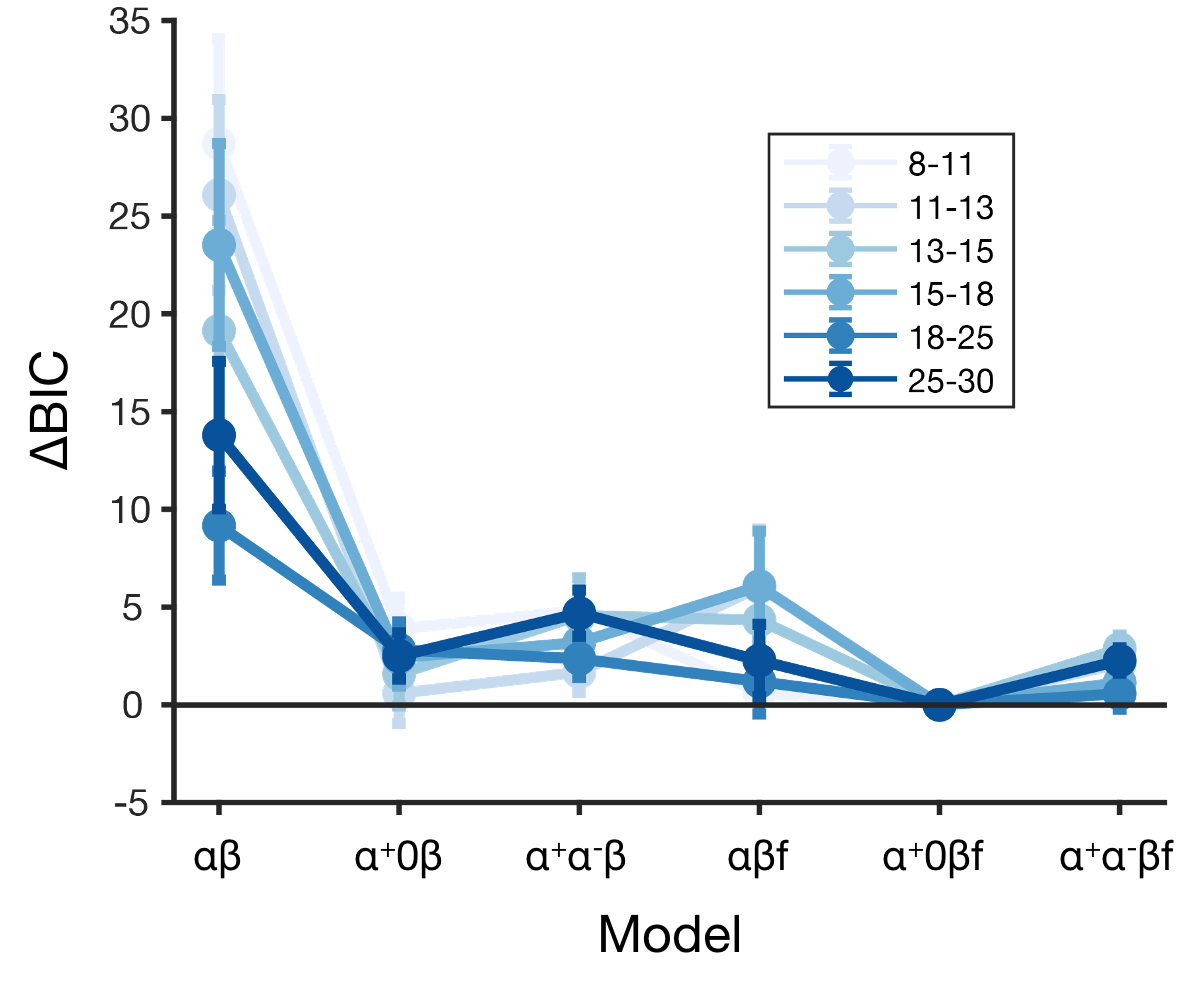

Supplement: S6 Fig — We calculated the difference between the BIC for each of the six models with α+0βf model per participant, represented by ΔBIC on the y-axis. Color represents 6 age groups. Results show that the α+0βf model is the winning model in all groups consistently. (TIF) [file pcbi.1008524.s007.tif]

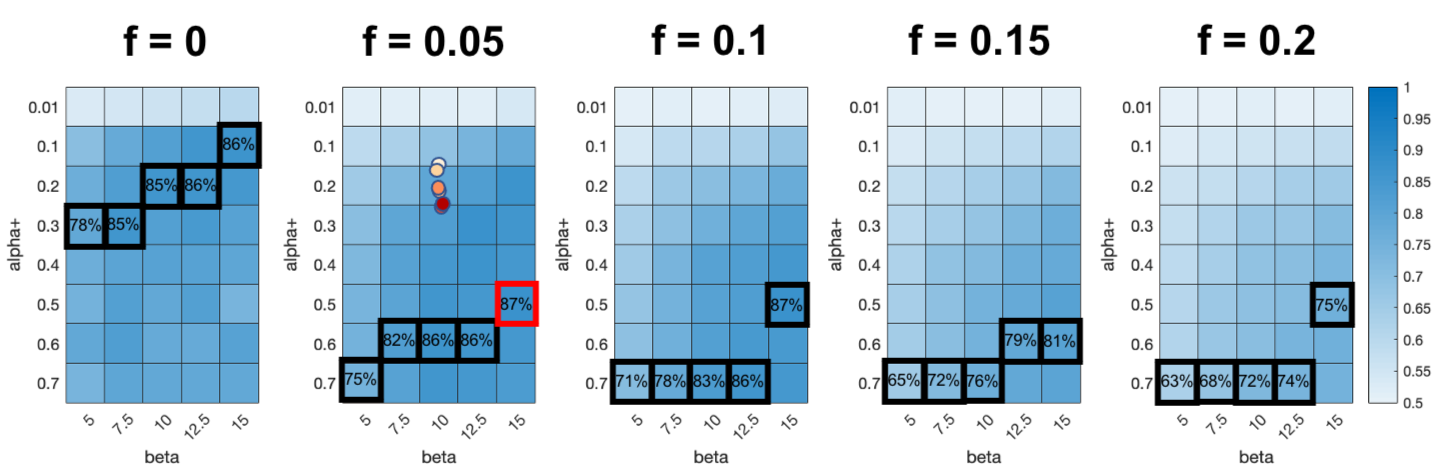

Supplement: S7 Fig — Overall simulated performance changes with respect to α+ (y-axis) and β (x-axis), where each subplot corresponds to f = 0 − 0.2 from left to right. Black rectangle highlights the local maximum within each column of each subplot (i.e. fixed β value), whereas the red rectangle highlights the global maximum. (TIF) [file pcbi.1008524.s008.tif]

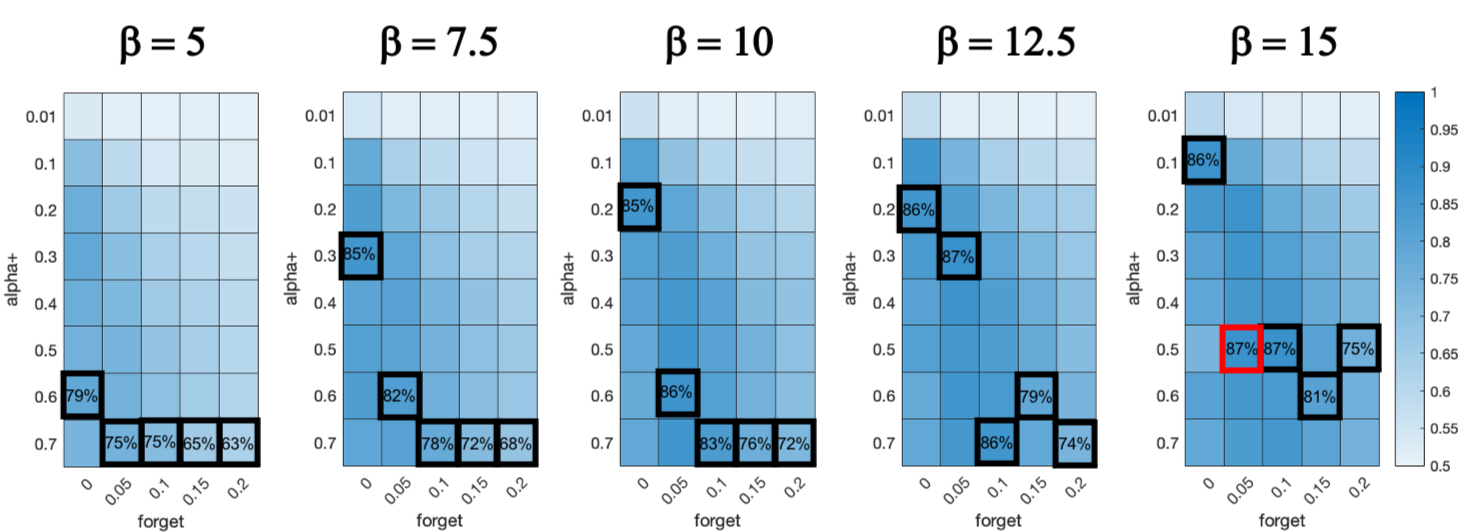

Supplement: S8 Fig — Overall simulated performance changes with respect to α+ (y-axis) and f (x-axis), where each subplot corresponds to β = 5 − 15 from left to right. Black rectangle highlights the local maximum within each column of each subplot (i.e. fixed f value), whereas the red rectangle highlights the global maximum. (TIF) [file pcbi.1008524.s009.tif]

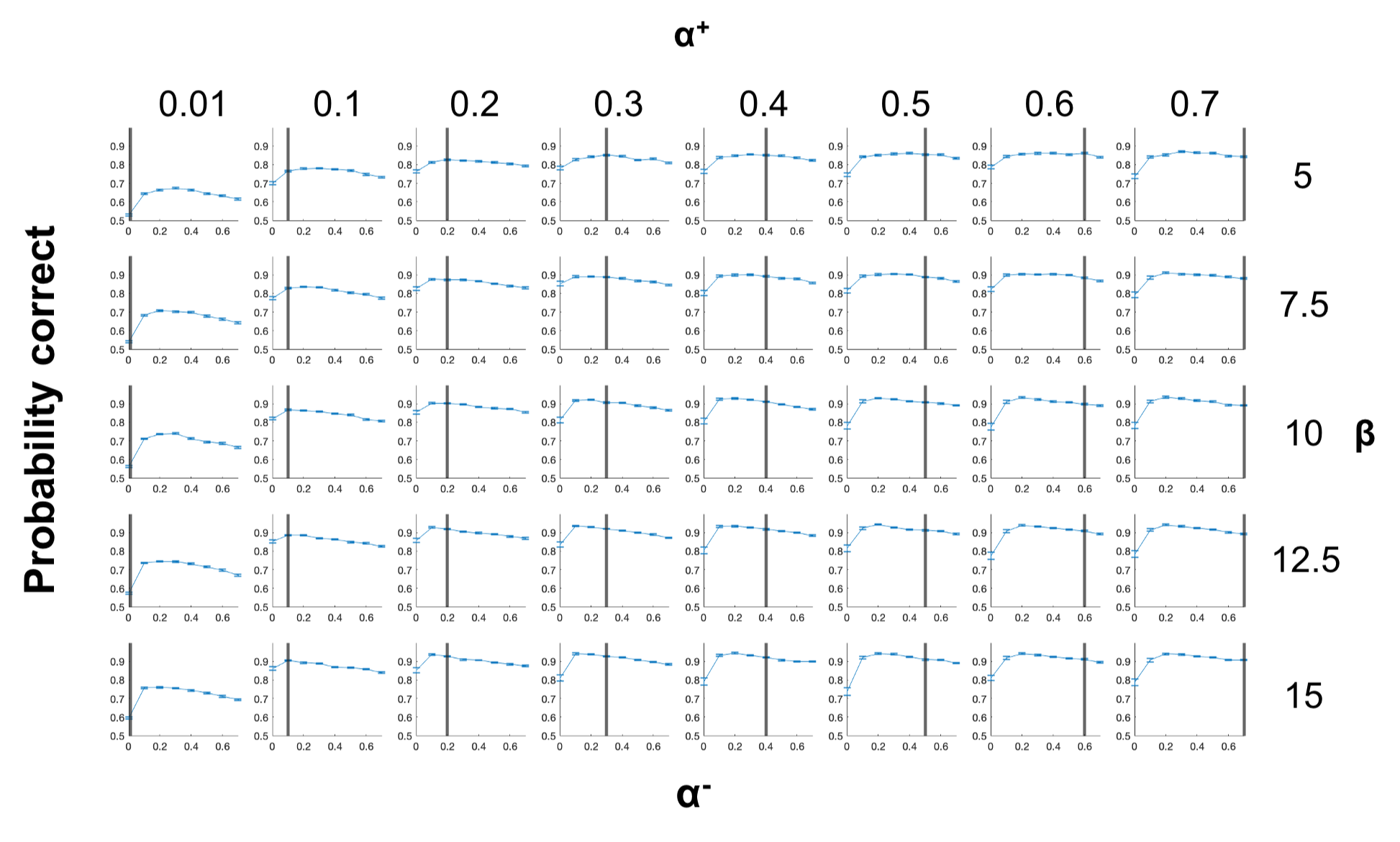

Supplement: S9 Fig — Overall simulated performance (y-axis) changes with respect to α− (x-axis), where each subplot corresponds to a combination of (α+, β) values. The vertical bar corresponds to the α+ value. The error bars show standard error across 100 simulations. (TIF) [file pcbi.1008524.s010.tif]

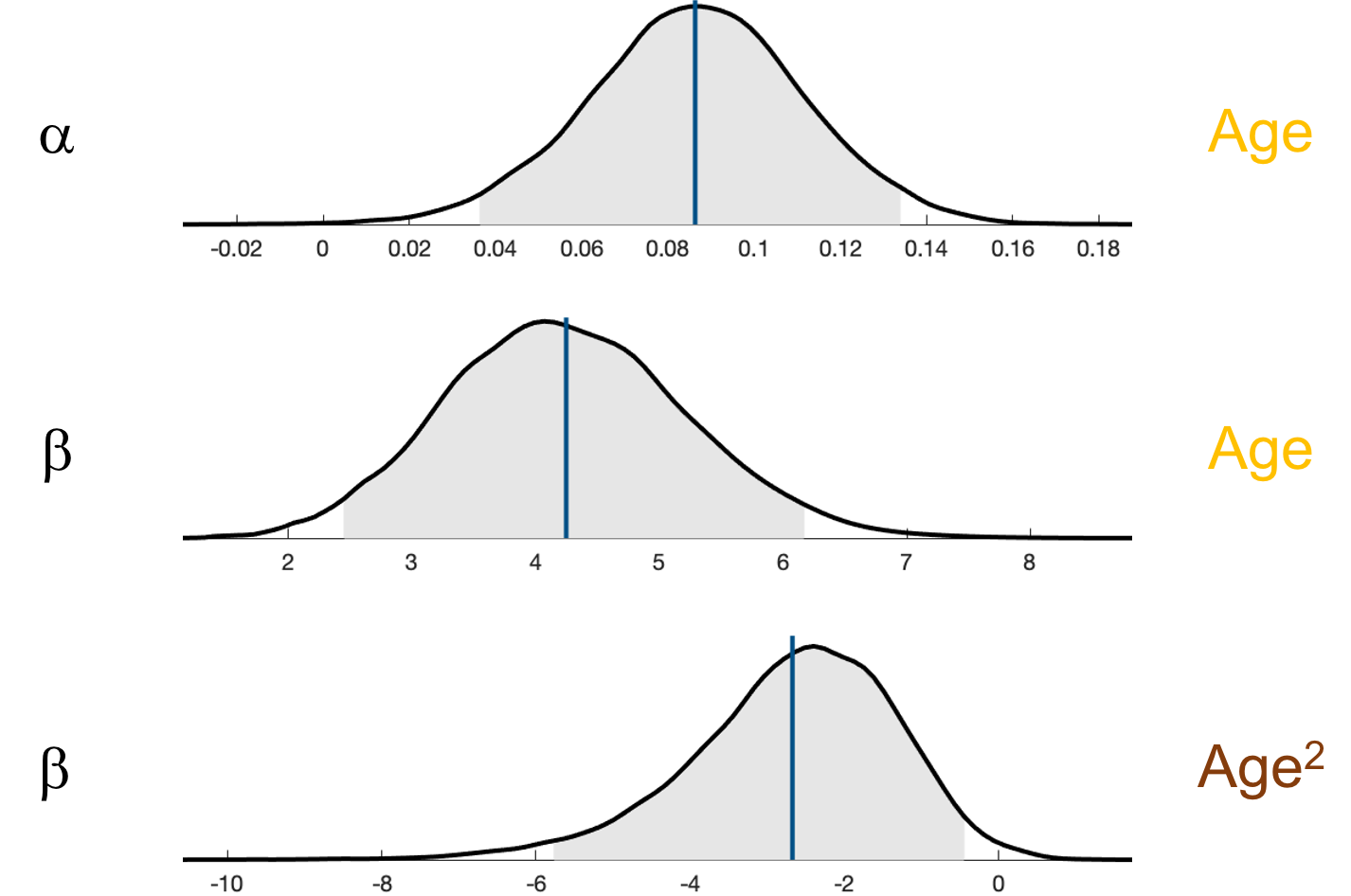

Supplement: S10 Fig — We directly incorporated age-related parameters into MCMC sampling to test within the hierarchical Bayesian modeling framework whether age had a linear or quadratic effect on the fitted parameters from the αβ model. We found positive linear effect of age on α and β, and negative quadratic effect of age on β. The model with quadratic age effect on α failed to converge. (TIF) [file pcbi.1008524.s011.tif]

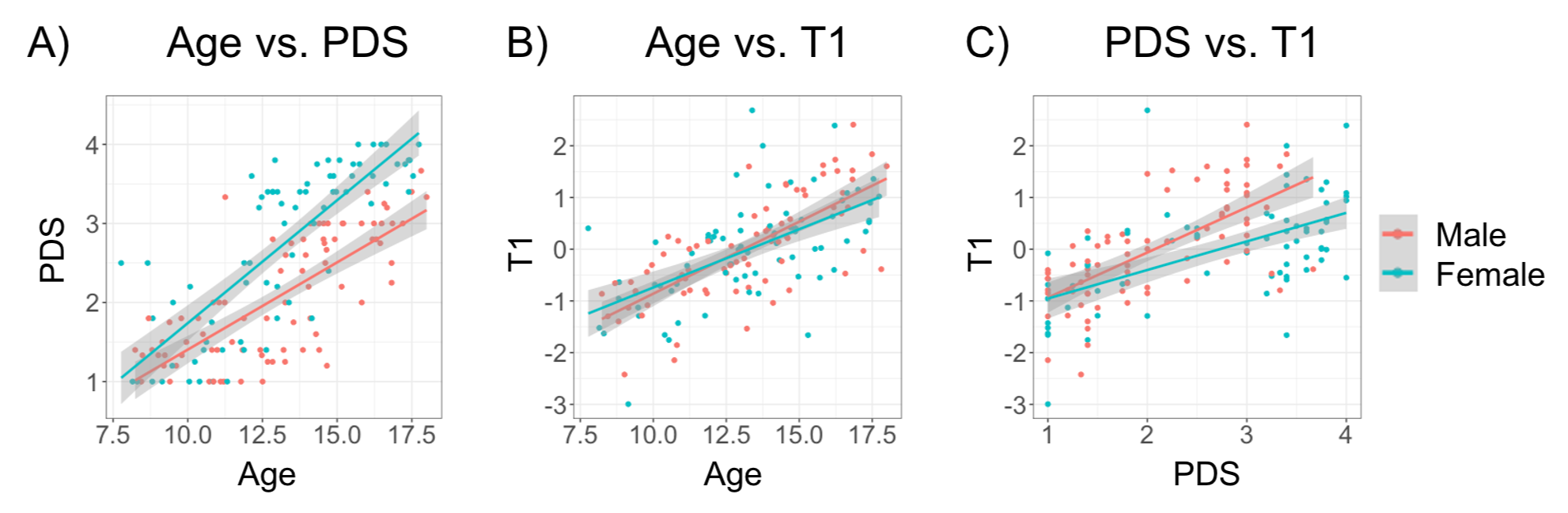

Supplement: S11 Fig — (TIF) [file pcbi.1008524.s012.tif]

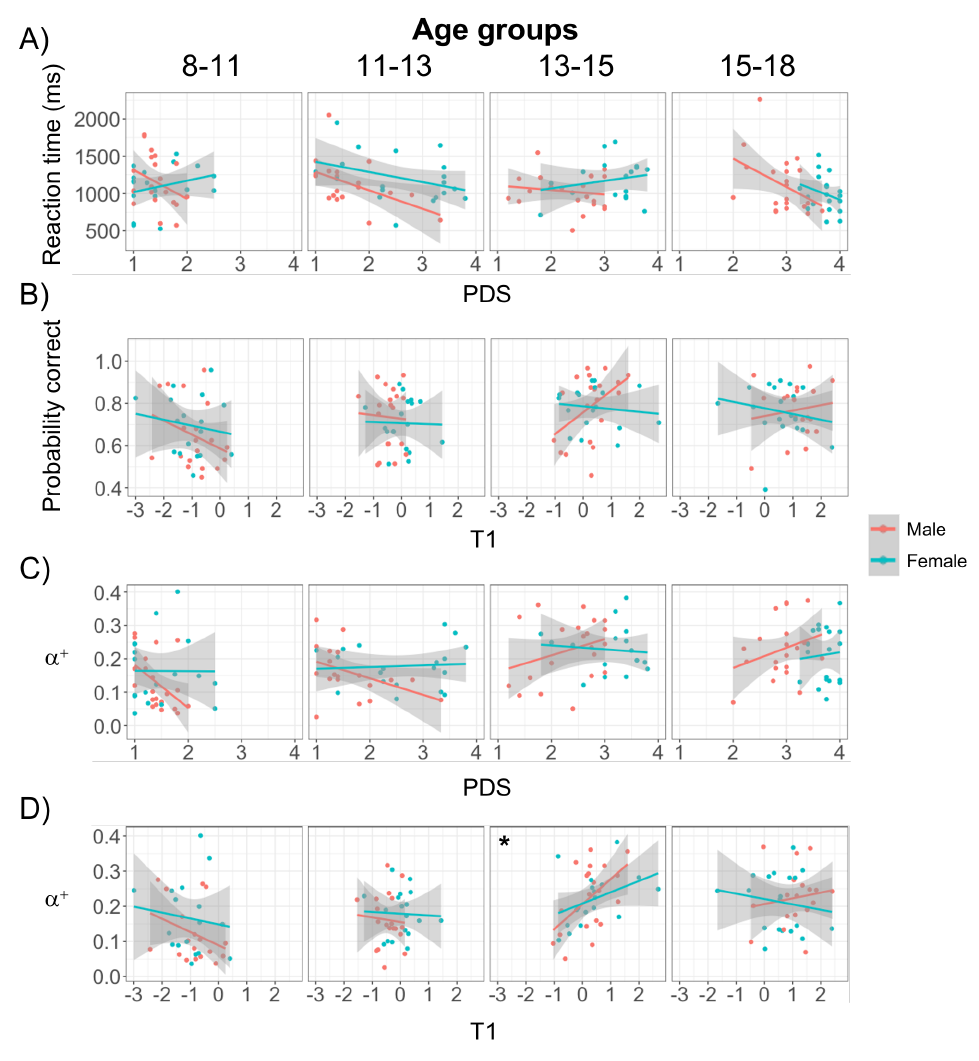

Supplement: S12 Fig — (A) PDS vs. median reaction time. (B) T1 vs. overall performance. (C) PDS vs. α+. (D) T1 vs. α+. (TIF) [file pcbi.1008524.s013.tif]

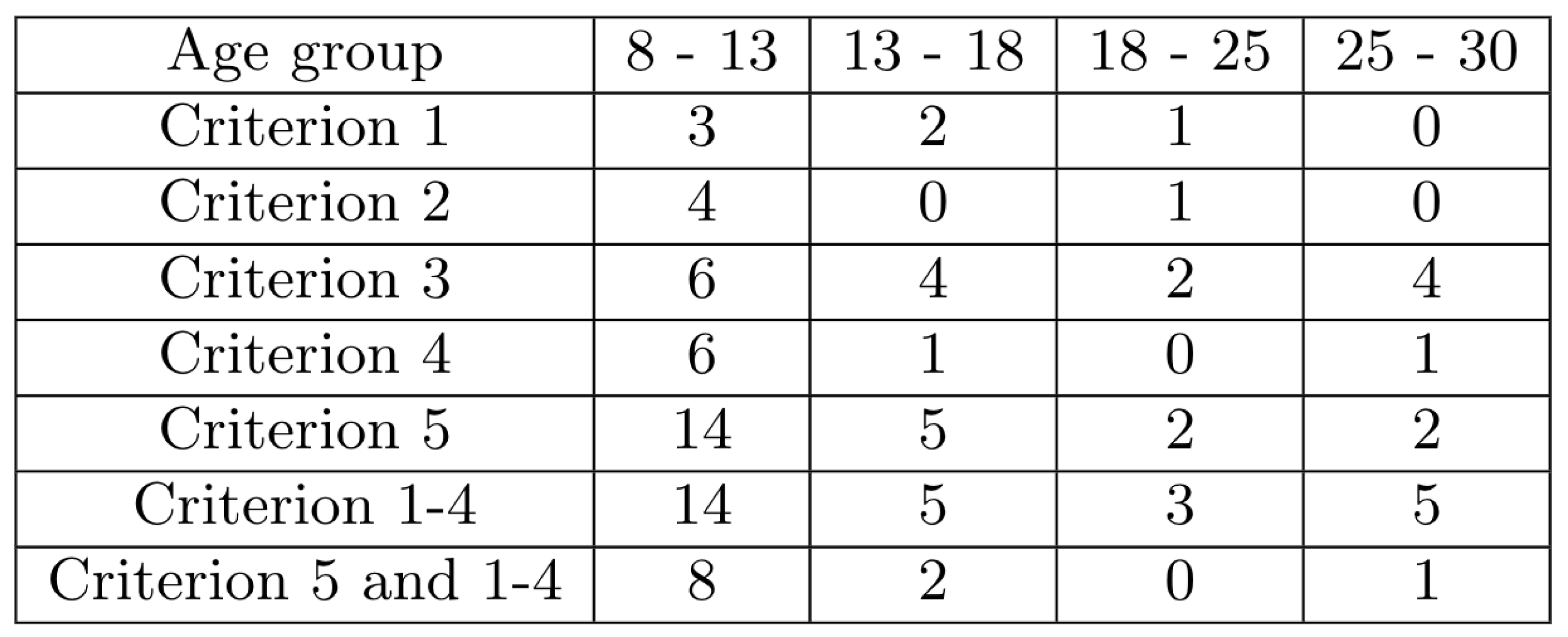

Supplement: S1 Table — (TIF) [file pcbi.1008524.s014.tif]

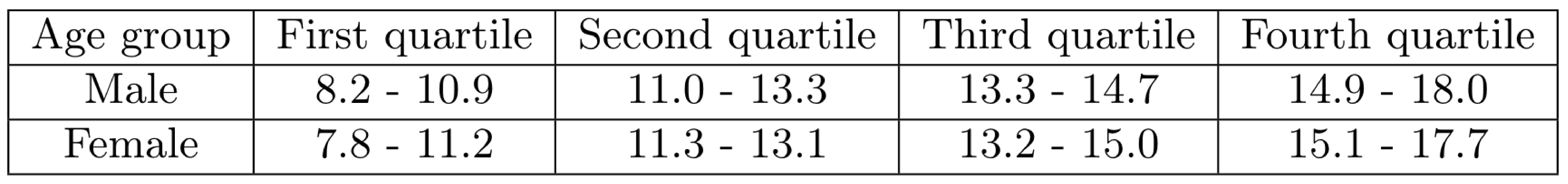

Supplement: S2 Table — (TIF) [file pcbi.1008524.s015.tif]

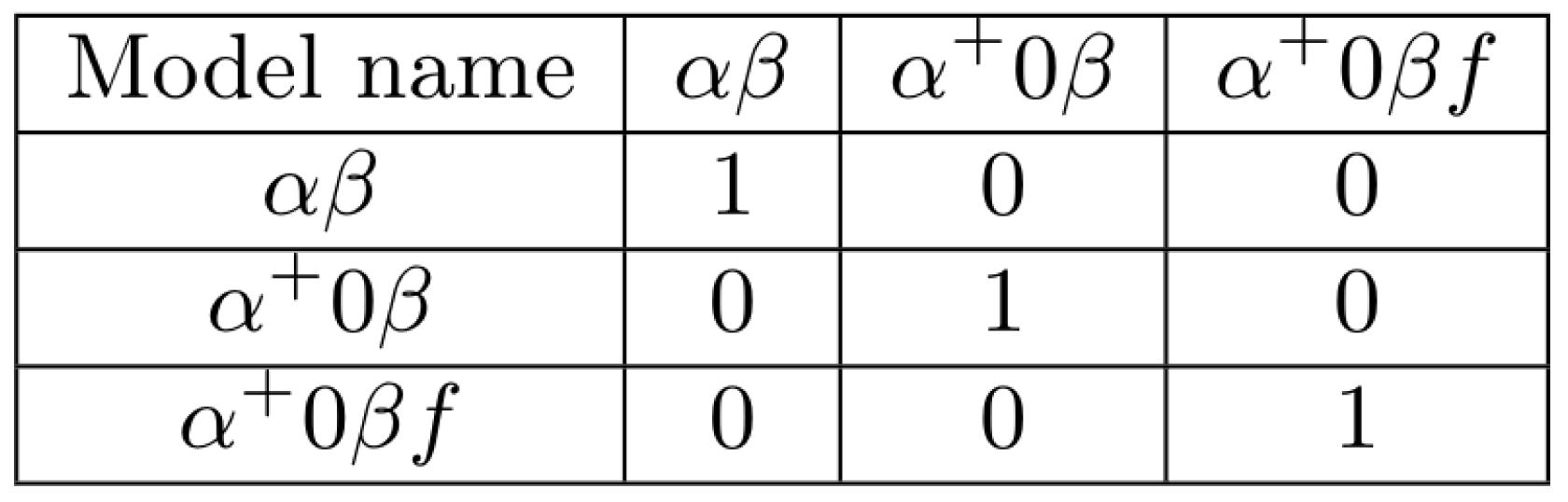

Supplement: S3 Table — The rows indicate the model where the dataset was generated from, whereas the columns indicate the model used for recovery. Each entry indicates protected exceedance probability. (TIF) [file pcbi.1008524.s016.tif]

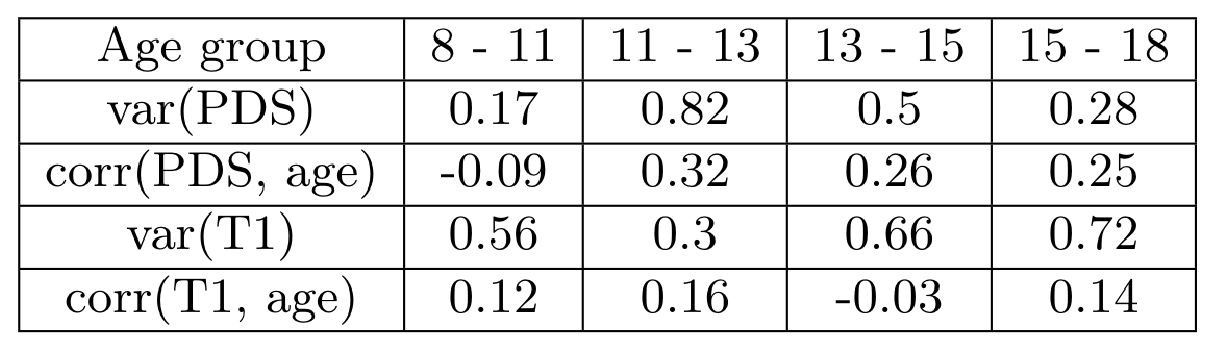

Supplement: S4 Table — Within each of the age group under 18, we calculated the variance of pubertal measures (PDS and T1) and their correlations to age. (TIF) [file pcbi.1008524.s017.tif]
